# 3D *in vitro* blood–brain barrier models: recent advances and their role in brain disease research and therapy

**DOI:** 10.3389/fphar.2025.1637602

**Published:** 2025-10-07

**Authors:** Laura O’Halloran, Olutoyosi Akinsete, A. Leah Kogan, Michelle Wrona, Amira F. Mahdi

**Affiliations:** ^1^ School of Medicine, University of Limerick, Limerick, Ireland; ^2^ Limerick Digital Cancer Research Centre, University of Limerick, Limerick, Ireland; ^3^ Health Research Institute, University of Limerick, Limerick, Ireland

**Keywords:** blood-brain barrier, 3D, *in vitro*, central nervous system, brain tumour, organ-on-a-chip

## Abstract

The blood-brain barrier (BBB) is a dynamic and highly selective interface crucial to central nervous system (CNS) homeostasis, presenting a major challenge for effective drug delivery in treating CNS pathologies such as brain tumours and neurodegenerative disease. Traditional two-dimensional (2D) *in vitro* models and animal models often fail to replicate the structural complexity and physiological functions of the human BBB. Recent advances in three-dimensional (3D) *in vitro* modelling offer enhanced physiological relevance by integrating cellular architecture, extracellular matrix (ECM) components, and dynamic fluid flow to simulate *in vivo* conditions more accurately. This review explores the structural and functional features of the BBB and highlights the evolution from 2D to 3D *in vitro* models, including hydrogel-based systems, microfluidics, organ-on-a-chip (OOAC) platforms, spheroids and organoids. The advantages of these models in recapitulating BBB dynamics and their application in cancer research and other CNS diseases are discussed. Finally critical comparison and discussion of current 3D models is presented, highlighting differences and best potential uses of each variation. Continued advancements are needed to develop accurate 3D *in vitro* models of the BBB in order to revolutionize drug screening, predict therapeutic efficacy, and support personalized medicine approaches. By providing robust, human-relevant platforms, 3D BBB models can accelerate drug development and treatment for patients affected by CNS pathologies.

## 1 Introduction

The blood-brain barrier (BBB) is a critical structure of the central nervous system (CNS). Lining the blood vessels of the brain, the BBB is responsible for environmental regulation, maintaining CNS homeostasis and protecting the brain from potential neurotoxic harm. It achieves this by regulating the paracellular and transcellular transport of molecules, from the bloodstream into the brain (Haddad-Tóvolli et al., 2017; O’Brown et al., 2018). This selective permeability is maintained by a network of neurovascular cells, primarily specialised, tightly connected endothelial cells, along with pericytes and astrocytes, which form a semi-permeable barrier allowing essential nutrients and ions to pass, while blocking toxins and pathogens. The importance of this barrier is underscored by the limited regenerative capacity of mature neurons; damage from neurotoxins can lead to irreversible neuronal loss (Abbott et al., 2010). However, the same selectivity that protects the brain also hinders drug delivery, especially for chemotherapeutic agents or larger antibody-based compounds (Angeli et al., 2019). This presents a major challenge in treating CNS diseases such as glioblastoma (GBM), where effective therapy depends on sufficient drug accumulation in brain tissue (Narsinh et al., 2024; Kadry et al., 2020).

Comprehending the structure and function of the BBB is necessary for drug development and delivery methods. However, studying the human CNS is difficult due to the invasive nature of procedures and lack of access to tissue. Whereas systematic drug distribution can be assessed using a simple blood sample, penetration to the CNS requires sampling of cerebrospinal fluid (CSF) or CNS tissue, which requires specialist skills and confers more discomfort, fear, risk and recovery time for the patient or research subject (Umemura et al., 2022). As such the development of pre-clinical models of the CNS and the BBB are imperative to the study of associated diseased and the creation of effective treatment options.

Many two-dimensional (2D) and three-dimensional (3D) *in vitro* BBB models have been developed to address the challenge of studying the BBB. Traditional 2D cell culture models are simple and cost-effective, however they often fail to accurately replicate the complex microenvironment of the BBB, limiting the understanding of drug permeability and efficacy (Tran et al., 2022; Williams-Medina et al., 2021). In contrast 3D *in vitro* models provide a more physiologically relevant platform by simulating the spatial and functional characteristics of the BBB (Chaulagain et al., 2023; Kaisar et al., 2017). This paper will review current 3D BBB models, their advantages over traditional 2D systems, and their applications in cancer and other CNS disease research, emphasizing their potential to enhance drug development and therapeutic innovation.

## 2 Blood-brain barrier (BBB)

The BBB was first identified in the 1800s, through Paul Ehrlich’s experiments that observed exclusion of intravascularly injected dye from the CNS tissues (Ehrlich). Since, the BBB has been extensively characterized in terms of structure and components. The BBB is centrally positioned within the neurovascular unit (NVU), a collection of neurons, pericytes, astrocytes, and microglia which interact with brain microvascular endothelial cells (BMECs) to couple cerebral blood flow to local neuronal activity (O’Brown et al., 2018; Keaney and Campbell, 2015; Sweeney et al., 2018a).

### 2.1 Brain microvascular endothelial cells (BMECs) and tight junction (TJ) proteins

**FIGURE 1:**
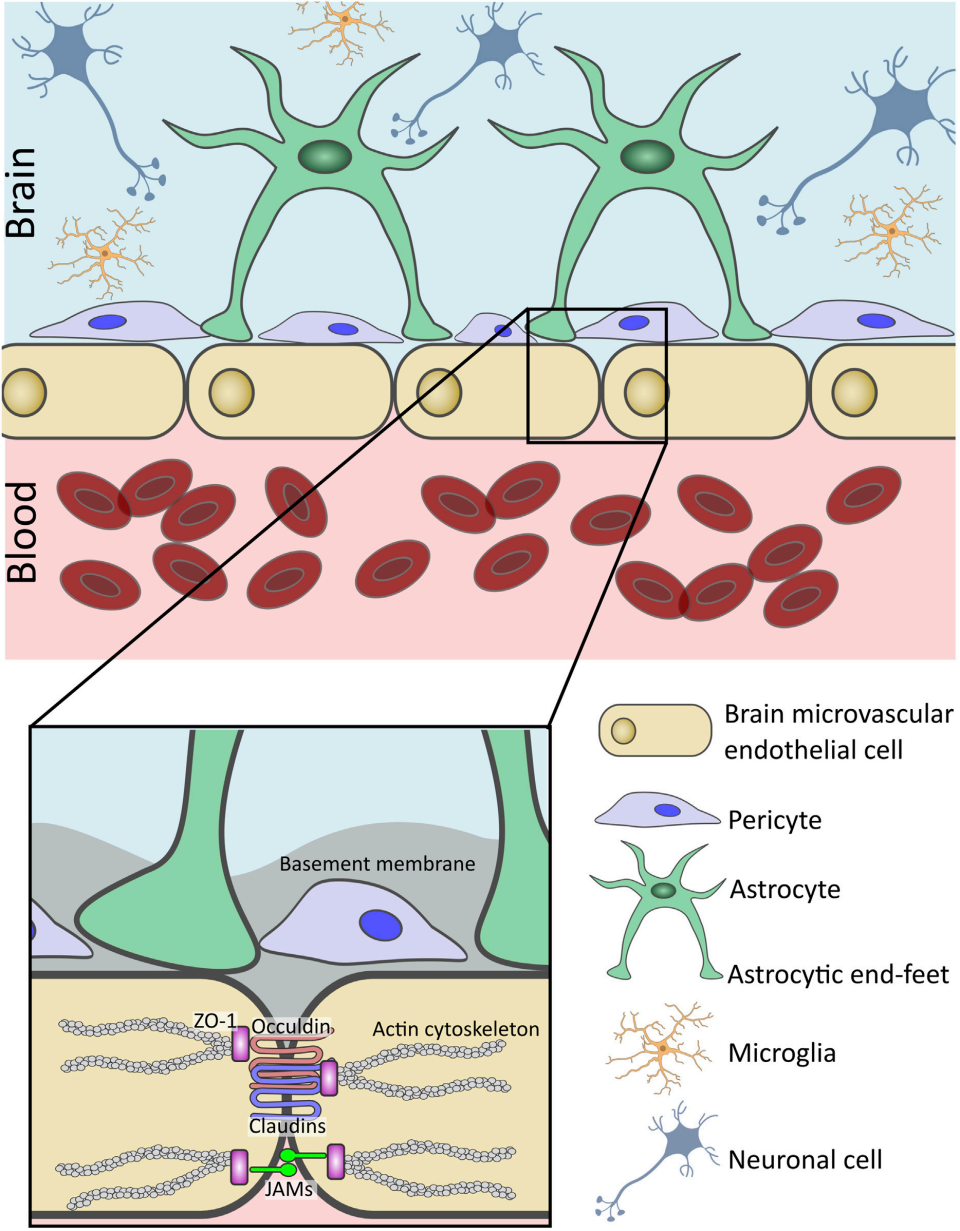
Structure and cellular components of the human blood-brain barrier (BBB). The BBB is a selective interface which regulates the passage of molecules from the bloodstream into the brain parenchyma. Linning the brain microvasculature, brain microvascular endothelial cells (BMECs) form the basis of the BBB. Connected by tight junctions (TJs) and junctional adhesion molecules (JAMs), they help to restrict paracellular transport across the BBB. BMECs are surrounded by a basement membrane (BM) and closely associated with pericytes also embedded within the basal lamina. Additionally, astrocyte end-feet wrap around the abluminal surface of the BMECs. Additional neurovascular unit (NVU) cells such as microglia support the function of the BBB and maintain central nervous system homeostasis.

The central unit of the BBB are BMECs, which form a physical barrier at the blood-brain interface (Figure 1). Unlike peripheral endothelial cells, BMECs are connected by tight junction (TJ) proteins, which tightly seal the paracellular space. This seal creates a selective barrier, allowing essential nutrients and gasses to pass into the brain, while restricting the movement of polar solutes or macromolecules (Abbott et al., 2010) (Figure 1, inset). TJs are comprised of proteins spanning the intercellular space, including occludin, claudins (CLDNs), and junctional adhesion molecules (JAMs). (Zhao et al., 2015; Abbott, 2013; Kubotera et al., 2019). Cytoplasmic zonula occludins (ZO) connect TJs to the cell’s cytoskeleton, ensuring BBB structural stability (Greene et al., 2019). Also found within the brain endothelium are adherens junctions (AJs), which bridge the intercellular space between BMECs and provide structural support to the BBB (Abbott, 2013). AJs are comprised of proteins such as cadherins and platelet endothelial cell adhesion molecule-1 (PECAM-1) and nectin (Zhao et al., 2015). Cadherins are anchored to the cell cytoplasm and cytoskeleton by α-, β-, and γ-catenin scaffolding proteins (Abbott et al., 2010).

Further distinguishing BMECs from their peripheral counterparts, is the absence of fenestrations and minimal pinocytic activity, additionally contributing to the low permeability of the BBB (Kadry et al., 2020). However, the CNS tissue has a strong demand for nutrients to support brain function. This is accomplished through specialized transport systems: solute carrier (SLC) transporters mediate the uptake of small molecules such as glucose, amino acids, and hormones, while larger molecules such as peptides and growth factors, are internalized via receptor-mediated transcytosis (RMT) (Nguyen et al., 2021; Ronaldson and Davis, 2022). Key RMT pathways involve the transferrin receptor (TfR), insulin receptor (IR), and low-density lipoprotein receptor-related protein 1 (LRP-1) (Baghirov, 2025). These receptors bind circulating ligands on the luminal membrane of BMECs, triggering clathrin-mediated endocytosis, vesicular transport and eventual exocytosis across the abluminal surface of the BMECs and into the brain (Haqqani et al., 2018a). Increasingly, these pathways are being explored for therapeutic delivery, as targeting RMT receptors offers a strategy to shuttle drugs into the brain with high specificity and efficiency (Pulgar, 2019; Haqqani et al., 2024). Antibodies, nanoparticles or other drug delivery systems can be engineered to recognise and bind to these receptors at the BBB (Haqqani et al., 2024; Haqqani et al., 2018b; Johnsen et al., 2017; Cepparulo et al., 2024). For instance, Song et al., 2022, developed a micelle-based delivery system, including isoliquiritigenin (ISL), a neuroprotective drug, modified with Angiopep-2, an LRP-1 targeting ligand, to improve the solubility and bioavailability of ISL, for the treatment of acute ischemic stroke (Song et al., 2022). In a mouse model, the modified ISL had increased accumulation within the brain, and reduced damage through cell autophagy inhibition and neuron death (Song et al., 2022).

BMEC transport systems also work to actively remove substances from the CNS. Drug efflux transporters, also known as ATP-binding cassette (ABC) transporters such as P-glycoprotein (P-gp) and Breast Cancer Resistance Protein (BCRP) are expressed on the luminal membrane of BMECs (Warren et al., 2009). ABC transporters maintain the exclusion of xenobiotics from the CNS by actively pumping substrates that have crossed the BBB back out into the peripheral blood stream. These transporters facilitate the removal of many environmental toxins and medications from the brain including, immunosuppressive, anti-inflammatory, antidepressant and psychotropic agents (Strazielle and Ghersi-Egea, 2015). Without these efflux transporters, many drugs could accumulate within the brain resulting in neural toxicity. For example, ivermectin, a known P-gp substrate, caused seizures and death in mice and P-gp-deficient dogs due to CNS accumulation (Schinkel et al., 1994; Mealey et al., 2001; Banks et al., 2024). P-gp and BCRP have a wide range of substrates, including chemotherapies like taxanes and small molecule inhibitors which can present a challenge when treating CNS diseases. Although they may be effective in treating primary tumours, the action of targeted therapies in the brain can be severely hindered by the efflux action of ABC transporters, as seen, for example, with ribociclib for metastatic breast cancer, or gefitinib for metastatic lung cancer (Sorf et al., 2018; Martínez-Chávez et al., 2019; Agarwal et al., 2010). Thus, overcoming or bypassing efflux transport mechanisms is a major focus in developing treatments for CNS diseases.

### 2.2 Pericytes

Pericytes are closely associated with BMECs in the CNS, as seen in Figure 1, and control the contractility of the CNS microvasculature (O’Brown et al., 2018; Arvanitis et al., 2020; Manu et al., 2023). Located on the abluminal side of the endothelium, they wrap around the BMECs (Abbott et al., 2010; Arvanitis et al., 2020). BMECs and pericytes are separated by a shared basal lamina, but direct contact is permitted through gap junctions which create a “peg-and-socket” structure (Abbott et al., 2010; Zhao et al., 2015; Ben-Zvi et al., 2014; Bernacki et al., 2008). Pericytes play a considerable role in maintaining integrity of the BBB. They contribute to the formation of TJs, by secreting molecules such as transforming growth factor-beta (TGF-β), which activates endothelial receptors, triggering pathways that produce TJ proteins like CLDNs and occludins (Trost et al., 2016; Jo et al., 2013). Furthermore, studies using pericyte deficient murine models, display increases in permeability of tracer dyes into CNS tissue (Armulik et al., 2010; Daneman et al., 2010). *In vitro* studies have also demonstrated the importance of pericytes, with their inclusion influencing ability of leukocytes to cross the BBB in simulations of CNS inflammation such as sepsis (McCloskey et al., 2024).

### 2.3 Astrocytes

Astrocytes are the most abundant cell type in the CNS and act as sensors for metabolic homeostasis (Keaney and Campbell, 2015; Arvanitis et al., 2020; Manu et al., 2023; Bernacki et al., 2008). As seen in Figure 1, their end-feet permit interaction with BMECs, by reaching into the perivascular space and enveloping majority of the abluminal surface of the capillary endothelia (Abbott et al., 2010; Keaney and Campbell, 2015; Arvanitis et al., 2020). Astrocytes support the BBB by releasing paracrine signalling molecules such as angiopoetin-1 (ANG1) and TGF-β which help maintain BBB integrity by promoting expression of BMECs junctional proteins and protecting against cell death (Manu et al., 2023; Fu et al., 2021). Astrocytes express a variety of transporters which regulate the passage of substances in and out of the CNS which would otherwise be prohibited by the BBB. For instance, glucose transporter 1 (GLUT-1), which regulates the uptake of glucose into the brain (Zhang et al., 2023), and glutamate transporters, glutamate transporter-1 (GLT-1) and glutamate aspartate transporter 1 (GLAST), which aid in regulating synaptic signalling through the glutamate-glutamine cycle (Andersen and Schousboe, 2023). Additionally, astrocytic end-feet connect through tight and gap junctions to form a secondary CNS barrier, the glia limitans (GL) (Arvanitis et al., 2020). The GL may act as a secondary protective barrier following failure of the BBB due to traumatic injury or neuroinflammation (Mora et al., 2020; Zhou et al., 2023). It is well established that astrocytes contribute to BBB integrity *in vivo* and *in vitro*, with early BBB models demonstrating the influence of astrocyte conditioned media in increasing trans-epithelial resistance and decreasing paracellular transport (Rubin et al., 1991). On the other hand, astrocytes, as the sensors of the NVU, can become reactive to inflammation caused by injury or disease, subsequently releasing factors than can increase BBB permeability and drive the BBB dysfunction seen in neurodegenerative disorders (Manu et al., 2023; Kim H. et al., 2022).

### 2.4 Microglia

Microglia are the most abundant innate immune cell-type in the NVU (Abbott, 2013; Arvanitis et al., 2020) and although not directly part of the BBB, they play a regulatory role (Figure 1). Once activated, microglia have two active states in which they can function to either promote BBB disruption or repair. In the M1 pathway, they release proinflammatory cytokines such as interleukin-1β (IL-1β) and tumour necrosis factor- α (TNF-α), thereby increasing BBB permeability. In the M2 pathway, they participate in tissue repair, phagocytosis of damaged cells and foreign substances, chemokine release, Vascular Endothelial Growth Factor (VEGF) release to promote blood vessel formation, and activation of neurotrophic pathways (O’Brown et al., 2018; Keaney and Campbell, 2015; Arvanitis et al., 2020).

### 2.5 Non cellular components of the BBB

BBB extracellular matrix (ECM) is highly specialised and can be found on both the luminal and abluminal surfaces. The vascular basement membrane (BM) is an ECM of structural proteins secreted by NVU cells (Keaney and Campbell, 2015). This 3D network is primarily made up of laminin, collagen IV, nidogen, and heparan sulphate proteoglycans such as perlecan (Xu et al., 2019). Measuring 20–200 nm in thickness, it serves to support the cells of the NVU and separate the abluminal surface of BMECs from neurons and glial cells (Figure 1, inset) (Thomsen et al., 2017). The BM supports BMECs, helping maintain BBB integrity and facilitating blood vessel development and maintenance (Thomsen et al., 2021). The BM facilitates many signalling processes within the vasculature and serves as a protective barrier against chemicals and cells attempting to penetrate the brain parenchyma. As observed in several neurological disorders, disruption of BM by matrix metalloproteinases is a crucial component of BBB dysfunction and leukocyte leakage (Daneman and Prat, 2015; Alahmari and Wu, 2021).

Finally, similar to peripheral endothelial cells, BMECs express a gel-like mesh layer of polysaccharides on their luminal cell surface, extending into the vascular lumen, known as the endothelial glycocalyx layer (Jin et al., 2021; Dancy et al., 2024). Composed of glycoproteins, proteoglycans, and glycosaminoglycans, primarily heparan sulphate, chondroitin sulphate, and hyaluronan, the glycocalyx plays a role in cell protection, adhesion, and signalling (Jin et al., 2021). Compared to peripheral vessels, the glycocalyx of the BBB is much denser and negatively charged (Profaci et al., 2020; Walter et al., 2021; A et al., 2018). The specific composition of the glycocalyx layer underscores its ability to act as a physical barrier between the blood and brain, a mechanosensor to modulate vasculature response to changes in shear stress, as well as playing a role in vasculature permeability and immune regulation (Dancy et al., 2024). The glycocalyx plays a role in maintaining CNS homeostasis, therefore, a disruption in its integrity can lead to increased BBB permeability, neuroinflammation, and the development of neurological diseases (Shi et al., 2025; Yang et al., 2021).

## 3 Rationale for studying the BBB

Neurological disorders are a major global health concern, leading to nervous system health loss in 3.4 billion individuals and 1.1 million deaths worldwide in 2021 (Steinmetz et al., 2024). Many of these conditions are linked to BBB dysfunction including, multiple sclerosis (MS), Alzheimer’s disease (AD), GBM and metastatic brain cancer (Arvanitis et al., 2020; Profaci et al., 2020). 22,439 M deaths occurred worldwide in 2019 (Qian et al., 2023). The global incidence of Alzheimer’s and other dementias increased by 147.95% from 1990 to 2019, rising from 2.92 million to 7.24 million cases (L et al., 2022). From the prospective of cancer demographics, GBM is the most frequently occurring primary malignant brain tumour, with an incidence of 3.23 cases per 100,000 people. Additionally, brain metastases (BrM) occur in 10%–40% of adult cancer patients, with the lungs being the most common primary site, responsible for about 50% of cases. (Qian et al., 2023; Lin et al., 2020; Pellerino et al., 2022; Mitchell et al., 2022). The global impact of CNS diseases underscores the critical need for developing effective therapies for affected patients.

Unfortunately, CNS targeted therapies are among the least approved cohort of therapies, with only 10%–12% CNS targeted therapies being approved per decade since the 1980s (Chaulagain et al., 2023). Many clinical trials are long and costly due to various challenges including, unpredictable disease progression, lack of biomarkers for tracking, and a need for patient focused endpoints (Stephenson et al., 2023). Furthermore, BrM patients are typically excluded from clinical trials due to poorer survival outcomes, higher risks of toxicity and concerns about limited therapeutic BBB penetration (Tan et al., 2022).

To address this challenge, numerous pre-clinical models of the BBB have been developed, to screen potential therapeutic candidates for their ability to cross the BBB and reach target sites within the brain, prior to clinical trial testing. *In vivo* animal models are the gold-standard for therapeutic screening, as they can capture the complexity of the BBB as well as provide insight into pharmacokinetic, pharmacodynamic and immunological variation. However, *in vivo* studies are often expensive and time-consuming and also often lack high-throughput capabilities, slowing the drug discovery process (Raut et al., 2022; Sharma et al., 2023). In addition, animal models can have a high degree of discrepancy from the human phenotype, resulting in around 80% of animal tested drugs failing in clinical trials (Perrin, 2014). For example, there is a high level of interspecies variation in the expression of ABC transporters (Warren et al., 2009) and TJ proteins such as CLDN-5 (Hoshi et al., 2013) by BMECs. The development of many *in vitro* BBB models has aided in addressing certain shortcomings associated with *in vivo* drug screening such as cost and complexity. However, accurately replicating the physiological characteristics of the BBB within a controlled *in vitro* setting remains a significant challenge.

## 4 Challenges to studying the BBB *in vitro*

### 4.1 Pathophysiology of the BBB

**FIGURE 2:**
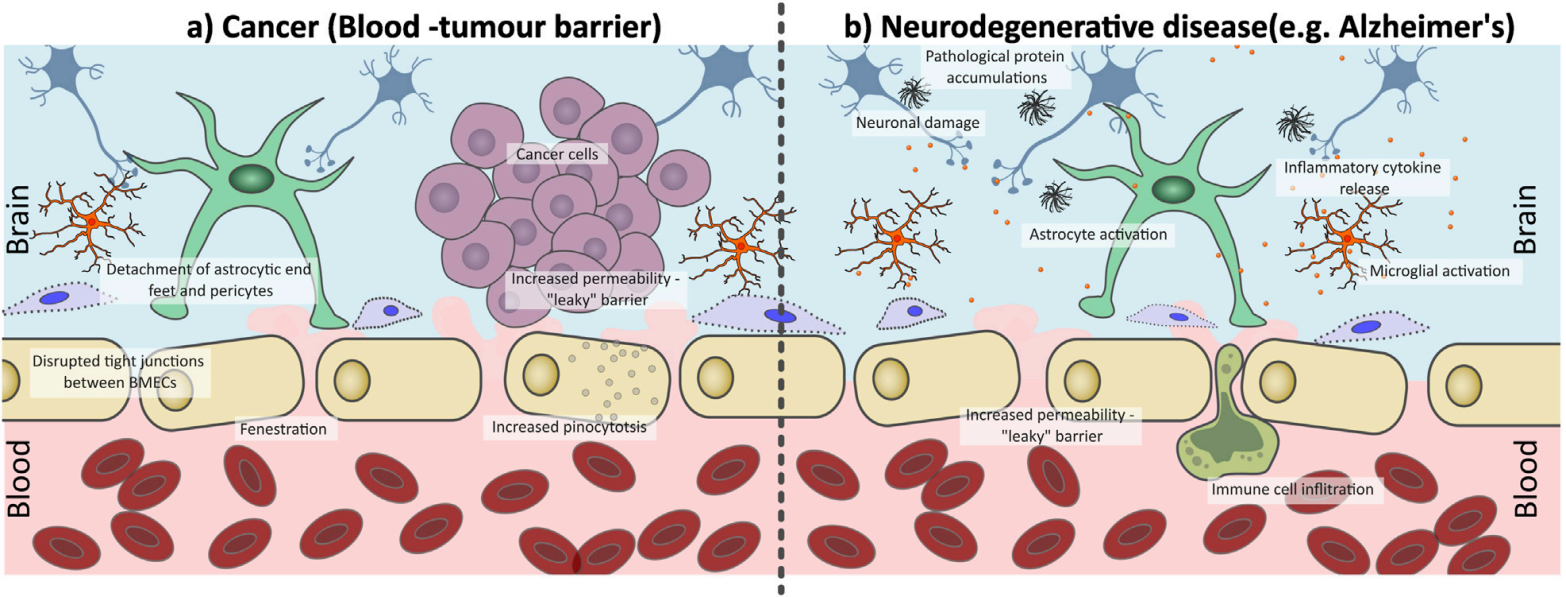
Disruption of the blood-brain barrier (BBB) under pathophysiological conditions **(a)** cancer and **(b)** neurodegenerative conditions. **(a)** In the presence of brain tumours the BBB is transformed into the blood-tumour barrier (BTB), which is characterized by abnormal, fenestrated endothelial cells with disrupted tight junctions (TJs) and increased pinocytosis activity, as well as a loss of pericyte and astrocyte interactions. This increases barrier permeability resulting in increased “leakiness” compared to a healthy functioning BBB. **(b)** Neurogenerative diseases such as Alzheimer’s Disease can trigger chronic inflation within the brain, therefore compromising the integrity of the BBB. There is a loss of endothelial cell interactions with astrocytes and pericytes, a loss of TJs which increases BBB leakiness. This allows for immune cell infiltration across the BBB, enhancing inflammation within the brain parenchyma, promoting disease progression.

The structural and functional properties of the BBB are shaped by both its cellular components and the surrounding microenvironment. However, capturing all characteristics of the BBB, such as intercellular communication, ECM interactions, and shear stress from blood flow, within a single *in vitro* model is extremely challenging. Given its dynamic nature, the BBB’s permeability can change in response to a range of conditions, including inflammation and disease, making it difficult to mimic (Sun and Song, 2024) (Figure 2). In cancer settings, remodelling of the BBB to the Blood-Tumour Barrier (BTB) occurs, leading to increased permeability. This often results from loss of TJs between BMECs, increasing paracellular transport across the BBB (Wolburg et al., 2003). This increased “leakiness” may present as an advantage to drug delivery; however, it is often heterogeneous. A study by Lockman et al., 2010, on BrM drug uptake and BTB permeability in immune-compromised mice, found over 89% of BrM exhibited partial BBB compromise, though the extent varied. In BrM, the uptake of therapies like doxorubicin and paclitaxel was higher compared to normal brain tissue, but lower than in other tissues or metastases outside the brain. Only around 10% of the most permeable metastases accumulate enough of these medications to cause cell death (Lockman et al., 2010).

Inflammation associated with neurodegenerative disease can also influence BBB integrity. Initial inflammation can activate microglia and astrocytes to maintain the BBB integrity through interaction with the BMECs. However, in chronic inflammatory states, such as those triggered by neurodegenerative conditions like AD or Parkinson’s Disease, astrocytic endfoot contact with BMECs is lost, leukocytes are recruited across the BBB (Brochard et al., 2008), pericytes can degenerate and lose coverage of the BBB (Sengillo et al., 2013) and expression of TJ associated proteins can be lost (Knox et al., 2022) (Figure 2). This dysfunction in the BBB can cause a pro-inflammatory positive feedback cycle, further damaging the BBB and promoting disease progression.

In AD, chronic inflammation reduces the activity of amyloid-β-degrading enzymes and inhibits the microglial cells’ ability to break down amyloid-β plaques which are the causative factor of AD neurological symptoms (Chen et al., 2024; Michelucci et al., 2009; Krabbe et al., 2013). Additionally, M1 microglia secrete factors like C-C motif chemokine ligand 2 (CCL2), TNF-α, IL-1β, and Interleukin 6 (IL-6), which can reduce TJ expression in BMECs, increasing BBB permeability. Furthermore, extravasated plasma components, stimulate the release of inflammatory chemokines, activating astrocytes and microglia, worsening inflammation and disease progression (Chen et al., 2024; Stamatovic et al., 2005). MS, a chronic autoimmune inflammatory disease of the CNS is characterized by demyelination and inflammation caused by immune cell invasion of the BBB. TJs between BMECs are affected through the release of pro-inflammatory cytokines such as TNF-α and Interferon-gamma (IFN-γ), compromising the BBB. This facilitates transport of harmful substances and immune cells into the brain parenchyma, which then contribute to disease progression (Shimizu and Nakamori, 2024; Sweeney et al., 2018b).

### 4.2 Accuracy of cell lines used to model BMECs *in vitro*

As previously discussed, evaluating potential therapeutics for treating CNS associated diseases is often challenging due to a lack of clinical trials. Furthermore, animal models are often expensive and the applicability of their results to human patients can vary. As a result, there is a significant interest in developing cell-based mimetics of the BBB’s *in vivo* characteristics such as, transport proteins, TJs and immune cell movement (Helms et al., 2016; Tello et al., 2022; Monteiro et al., 2024). These cell-based models mainly utilize primary and immortalized cell lines from species such as humans, mice, rats and pigs. Compared to immortalized cells, primary cells often offer a more physiologically relevant model. Each cell line offers many advantages but also have certain limitations, specifically around barrier properties, which can in turn add to the challenge of studying the BBB (Monteiro et al., 2024; Jackson et al., 2019; Fujimoto et al., 2020). Table 1 Summarises the various cell lines used to create *in vitro* BBB models, emphasising key advantages and disadvantages. Inter-cell line and inter-species variability can hinder the reproducibility and translation of the results from *in vitro* BBB models, thus the choice of cell line when developing BBB models is integral to the model’s validity (Hoshi et al., 2013; Hirsch and Schildknecht, 2019).

**TABLE 1:** Summary of the cell types used in place of brain microvascular endothelial cells for *in vitro* modelling of the BBB.

Type	Example and research resource identifiers	Advantages	Disadvantages	References
Immortalised brain cell lines - Human	hCMEC/D3 (CVCL_U985)	• Maintains human BBB characteristics by expressing key TJ proteins (occludin, claudin-5), receptors, and transporters • Immortality ensures reproducibility and scalability	• Low junctional tightness, limiting use in small molecule transport studies for BBB permeability • Low (Trans-epithelial electrical resistance) TEER values • Require optimal culture conditions (e.g., removal of growth factors, exposure to shear stress) to maintain BBB-like properties, complicating experiments and reproducibility	Monteiro et al. (2024), Daniels et al. (2013), Weksler et al. (2013)
	HBEC-5i (CVCL_4D10)	• Retain many human BMEC characteristics • Immortality ensures reproducibility and scalability • Can grow without adjuvants, and are therefore easier to use routinely for screen drug interactions • High TEER values	• Improved functionality with astrocyte-conditioned media, suggesting a reliance on astrocytes to better mimic *in vivo* BBB characteristics • High TEER values may not be maintained over long periods of time	Monteiro et al. (2024), Puech et al. (2018)
Immortalised brain cell lines – non-human	RBE4 (CVCL_0495)	• Cost-effective and reproducible • Express TJ proteins, e.g., CLDNs • Express BBB transporters, P-gp, GLUT-1 and 3	• Does not replicate full *in vivo* human BBB due to species differences • Low expression of certain TJ proteins such as occluding • Low/no expression of transporters such as ABCG2 limiting their ability for studying specific drug interactions • Lacks a fully restrictive paracellular barrier, limiting use for screening small-molecule drugs	Reichel et al. (2002), Balbuena et al. (2010), Veszelka et al. (2018)
Primary brain cell line - human	Human Brain Microvascular Endothelial Cells (HBMECs)	• Closest match to human BBB. • Express TJ proteins, e.g., CLDNs, occuldin • Express BBB transporters, P-gp • Have high TEER values compared to other primary cultures	• Limited tissue availability/hard to culture • Have limited lifespan • Ethical concerns regarding obtaining tissue • High cost to purchase and culture	Monteiro et al. (2024), Stone et al. (2019), Bernas et al. (2010), Zhang et al. (2011)
Primary brain cell line- non-human	Rat brain microvascular endothelial cells (RBMECs)	• Mimic BBB characteristics such as TJ formation and selective permeability • Easily isolated • Cost-effective	• Does not replicate full *in vivo* human BBB due to species differences • Have limited lifespan	Stone et al. (2019), Watson et al. (2013), Burek et al. (2019)
Non-brain barrier forming cell lines - human	Caco-2 (CVCL_0025)	• Form tight monolayer with TJ expression • Do not require expensive media supplements • P-gp expression, enabling study of uptake mechanisms drug efflux • Cost-effective • Applicable to high-throughput screening	• Do not originate from the brain but from colon carcinoma	Veszelka et al. (2018)
Non-brain barrier forming cell lines – non-human	MDCK, MDCK-MDR1 (CVCL_0422)	• Forms tight monolayer with TJ expression • Do not require expensive media supplements • P-gp expression, enabling study of uptake mechanisms drug efflux • Cost-effective • Applicable to high-throughput screening	• Does not replicate full *in vivo* human BBB due to species differences • Do not originate from the brain but from canine kidney • Less relevant for BBB studies compared to Caco-2 or more specialized BBB models	Monteiro et al. (2024), Veszelka et al. (2018), Dukes et al. (2011)
Stem cells	Human-induced pluripotent stem cells (iPSCs)	• Can be differentiated into BMECs • Can be used to create complex co-culture systems • Patient-specific characteristics and pathologies can be captured • Derived from adult individuals therefore avoiding ethical concerns of using embryonic tissue	• iPSC differentiation into BMECs is often complex and expensive • Challenging to obtain from healthy individuals • Maintaining cultures is labour-intensive and requires specialized expertise which may not be available in all research settings	Delsing et al. (2020), Gaston-Breton et al. (2023)

Recent advances have shown an increased interest in human induced pluripotent stem cells (iPSCs), which have unlimited self-renewal capacities and can regenerate into a plethora of somatic cell types (Gonzales-Aloy et al., 2023). BMECs derived from iPSCs are often used when modelling the BBB *in vitro*. These cell types can be derived from various sources commercially or isolated from human samples (Vetter et al., 2025). BMECs, astrocytes and pericytes derived from iPSCs have shown to demonstrate cell-cell interactions and barrier functions similar to *in vivo* conditions, such as TJ and transporter expression, making iPSCs a novel cell type that can lead to strengthening *in vitro* models (Hajal et al., 2022; Yamaguchi et al., 2023). Additionally, iPSCs provide a possible approach for developing patient-specific BBB models that incorporate multiple neural cell types, mimetic of the neurological pathophysiology observed in CNS disorders (Osaki et al., 2018).

### 4.3 Assessing BBB model validity and integrity

When developing a physiological BBB model, it is essential to establish a reliable method for assessing barrier integrity. Understanding how potential therapeutics and treatment modalities interact with, and affect barrier integrity provides insight into their efficacy and suitability for treatment of CNS pathologies (Williams-Medina et al., 2021; Sun et al., 2021). Overall, assessing BBB integrity remains challenging due to the lack of standardized methodologies, which often fail to capture the full complexity of barrier dynamics. Measurement of barrier integrity and permeability can be achieved through various methods such as, trans-epithelial electrical resistance (TEER), or measurement of paracellular transport of tracer substances (Vetter et al., 2025). The expression of proteins and genes involved in TJ formation, such as ZO-1, can serve as indirect indicators of blood-brain barrier integrity.

**FIGURE 3:**
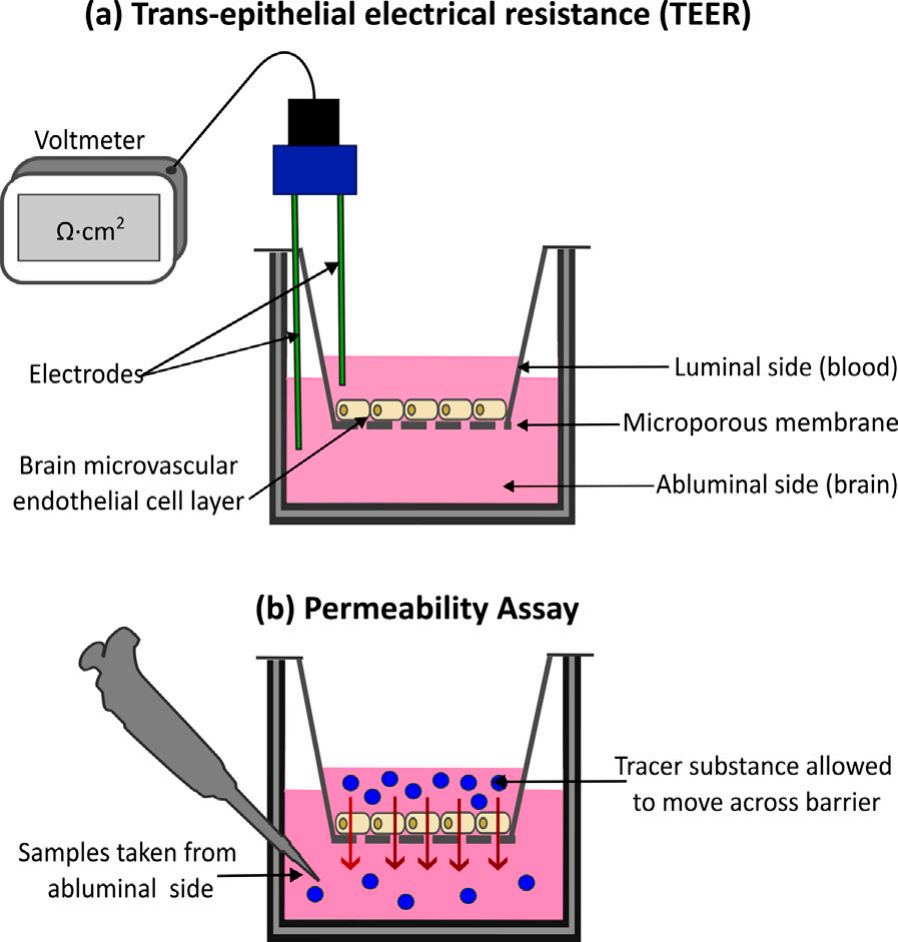
Methods for assessing blood-brain barrier (BBB) integrity and permeability *in vitro*. **(a)** Trans-epithelial Electrical Resistance (TEER) is a quantitative method for assessing barrier integrity. A small electrical current is applied to the barrier, while two chopstick electrodes, placed either side of the cell layer measure the resulting resistance. High TEER values indicate a strong intact barrier, with low values signifying compromised barrier integrity. **(b)** Tracer substances, such as fluorescently tagged molecules are also used to measure barrier integrity. Measuring the movement of the substance across the cell layer, example via spectrophotometry, can provide insight into the integrity of the barrier. Higher substance movement indicates increased barrier permeability, with low movement indicating a strong barrier.

TEER is a quantitative measure that serves as a gold standard for assessing BBB integrity non-invasively, repeatedly and with minimal impact on cell viability (Srinivasan et al., 2015; Malik et al., 2025). TEER works on the principle that charged ions must move across a cellular barrier through paracellular movement and the increased integrity of a barrier via TJ connections and cellular health will impede paracellular permeability. TEER is measured by applying a small electrical current across a cell layer, such as the epithelial layer seen in the BBB, using two chopstick electrodes placed on either side of the barrier (Figure 3a). The cell layer is subjected to a small alternating current which stimulates the movement of charged ions present in the cell media across the barrier in a paracellular fashion (Srinivasan et al., 2015). The high transport resistance of ions through a barrier generates electrical resistance, which can be measured via a voltometer, according to Ohm’s Law (Linz et al., 2020). The surface area of the cellular barrier (in cm^2^) is multiplied by the measured resistance (ohms Ω) to determine the TEER (Ωcm^2^) (Srinivasan et al., 2015). A high TEER reading is an indicator of an *in vitro* BBB with strong integrity and expression of functional TJs (Jackson et al., 2019; Orzeł- Gajowik et al., 2024).

There are limitations to the precision, accuracy and practicality of TEER measurements. Measured values can vary due to various biological and environmental factors including positioning of electrodes, device variability, cell discrepancies, culturing conditions, temperature, and TJ pathology (Srinivasan et al., 2015; Ghaffarian and Muro, 2013). Furthermore, traditional TEER measurements using chopstick electrodes are designed to measure barrier integrity of a monolayer. Due to this, measuring the integrity of more complex 3D models can be difficult. To evaluate TEER, these models may need to be disrupted to form a 2D monolayer (Warschkau et al., 2022). For microfluidic or organ-on-a-chip (OOAC) models, electrodes often need to be embedded directly into the device materials, which introduces challenges related to biocompatibility and material compatibility with the cells and culture environment (Malik et al., 2025; Nazari et al., 2023). Overall, such variations can limit the comparability of TEER assay data, making it difficult to consistently assess BBB model integrity across studies (Sharma et al., 2025).

The integrity of the BBB can also be assessed using permeability assays with tracer substances. The apparent permeability (*P*_app_) of these tracer substances across the cultured cellular barrier helps to reflect the tightness of the endothelial TJs, giving insight into the barrier’s integrity. The more substance that can travel across the barrier the weaker and “leakier” it is. (Thomsen et al., 2021; Appelt-Menzel et al., 2017; Rice et al., 2022) (Figure 3b). *P*_app_ is determined from the amount of tracer substance that travels across the cultured barrier within a particular length of time. It is calculated using the molecule steady-state flux, the surface area of barrier, and the substance’s initial concentration in the donor chamber (Vetter et al., 2025; Doryab and Schmid, 2022; Hubatsch et al., 2007). The amount of tracer substance that penetrated the cell layer is quantified using spectrophotometry or fluorescent microscopy (Sun et al., 2021). Various tracer substances can be utilized for permeability assays, the most popular being fluorescence-labelled dextrans with molecular weights ranging from 3–70 kDa. Others include sodium fluorescein (NaF), Evan’s blue, horseradish peroxidase, and lucifer yellow (Chaulagain et al., 2023; Vetter et al., 2025). However, unlike TEER, permeability assays are endpoint techniques and therefore cannot give insight into real-time dynamic barrier changes (Malik et al., 2025). Additionally, results obtained from permeability assays depend on the type, size, charge and properties of the tracer molecule (Miki et al., 2014). Permeability is also influenced by the complexity of the model, with co-cultures showing reduced permeability compared to monocultures (Vetter et al., 2025; Campisi et al., 2018; Seo et al., 2022).

Specific tracer substances can also be used to measure the functional activity of efflux transporters at the barrier such as P-gp and BCRP. Such assays do not inform of the permeability or integrity of the barrier, but rather the expression and activity of BBB specific proteins which contribute to the validity of the model and are used in tandem with TEER or permeability assays. A commonly used substrate tracer is rhodamine-123, which is a substrate of P-gp, that shows increased accumulation in the brain compartment of BBB models when cells are treated with a P-gp inhibitor such as cyclosporin A (Fontaine et al., 1996; Mittapalli et al., 2013).

Lastly, investigation of TJ and transporter protein and gene expression provides a method for evaluating the integrity and functionality of the BBB (Rice et al., 2022). For spatial resolution, key TJ proteins such as ZO-1, CLDN-5 as well as transporters such as P-gp can be fluorescently labelled and imaged using confocal microscopy to evaluate TJ organization and barrier function (Alluri et al., 2024).

## 5 2D *in vitro* BBB models

### 5.1 Monoculture Transwell

**FIGURE 4:**
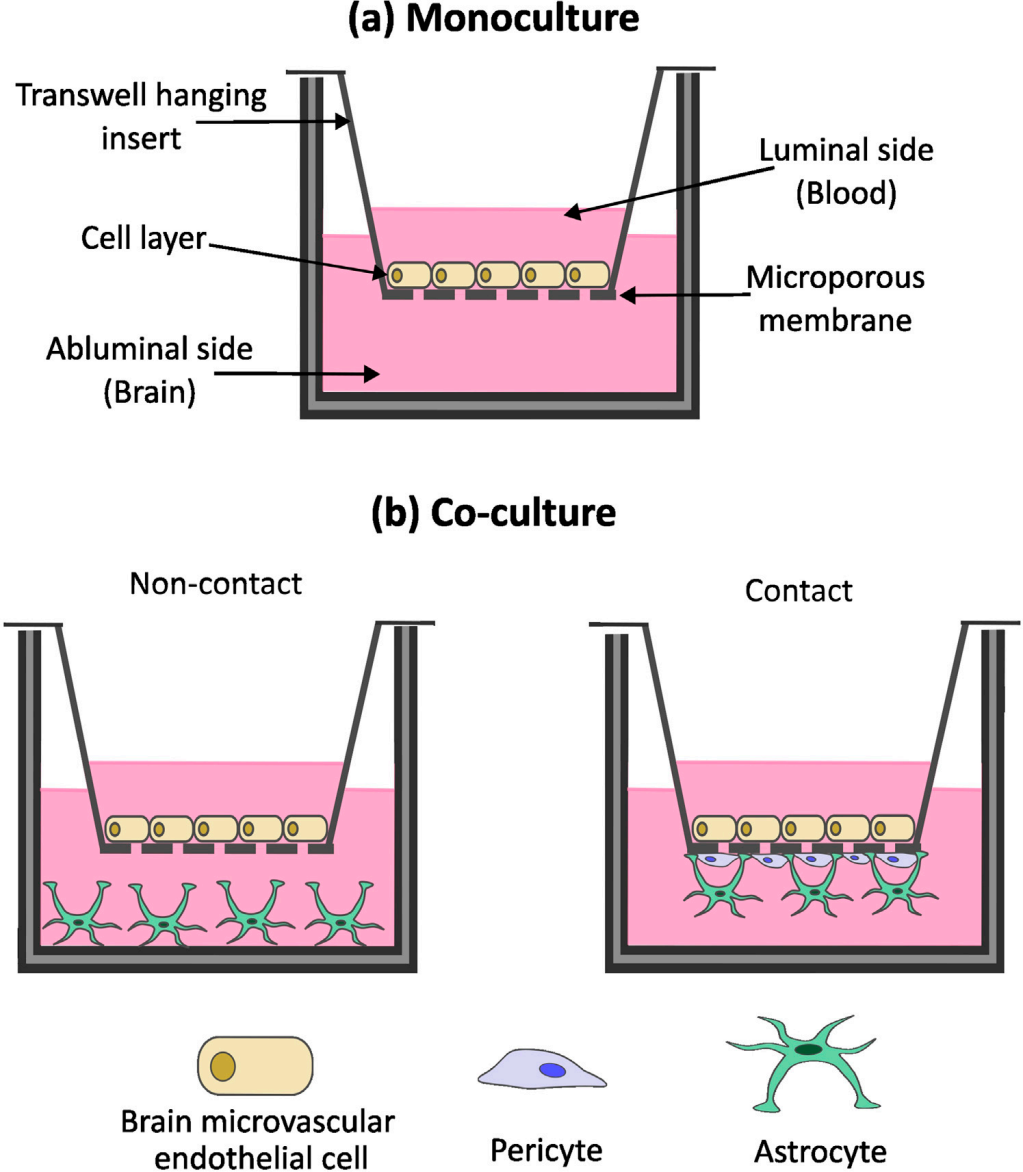
Two-dimensional (2D) *in vitro* models of the blood-brain barrier (BBB). **(a)** A Transwell inserts create an upper chamber with a semi-permeable membrane at its base, sitting into a well to create a bottom chamber. Monoculture *in vitro* BBB models are composed of a singular cell type, often brain microvascular endothelial cells (BMECs), cultured onto the semi-permeable membrane of a Transwell insert, representing the luminal side of the BBB, with the lower chamber representing the abluminal side. **(b)** Co-culture BBB models incorporate multiple cell types and can be categorized into non-contact or contact co-culture models. Non-contact models are comprised of endothelial cells cultured on top of the semi-permeable membrane, with additional NVU cell such as pericytes and astrocytes cultured within the bottom well. Contact cultures are comprised of endothelial cell cultures on top of the semi-permeable membrane and additional NVU cells cultured directly onto the of the insert, increasing cell-cell contact.

Transwell systems are used extensively to model the BBB *in vitro* (Williams-Medina et al., 2021; Ghaffarian and Muro, 2013; Santaguida et al., 2006) (Figure 3). The simplest of this model was initially employed for monocultures of barrier forming cell lines such as Caco-2 or BMECs (Jackson et al., 2019; Stebbins et al., 2016). Monoculture Transwell BBB models offer a simplified platform to investigate barrier properties in a controlled environment, making them advantageous for initial drug screening. Cells are cultivated on to the microporous membrane or filter of a Transwell insert and allowed to grow and differentiate into a continuous monolayer (Figure 4a). This upper Transwell insert represents the luminal side or “blood” of the BBB, while the surrounding well represents the abluminal or “brain” side (Rice et al., 2022; Stebbins et al., 2016; Sivandzade and Cucullo, 2018; Stone et al., 2019). By separating the luminal and abluminal sides, this model allows for the exchange of solutes while restricting the movement of cells (Wilhelm and Krizbai, 2014). Transwell filter membranes can differ in porosity and composition and can also be coated with materials to improve cellular adhesion and growth such as ECM proteins (Williams-Medina et al., 2021).

### 5.2 Co-culture transwell

As the understanding of the BBB developed, it was noted that other cells of the NVU contributed significantly to the formation and integrity of the BBB, not just the BMECs. This was observed via the inclusion of other cell types in the Transwell model, in a co-culture format, including astrocytes, pericytes, microglia and neurons. Co-culture of other NVU cells with BMECs showed numerous advantages in creating a more physiologically relevant model including: increase in TEER values; decrease in paracellular permeability; increased expression of BBB-associated transporters and increased formation of TJs, as reviewed thoroughly in (Monteiro et al., 2024). Co-culture BBB models can be categorized into two groups: the contact co-culture and the non-contact co-culture (Sivandzade and Cucullo, 2018; Blanchard et al., 2020; Kulczar et al., 2017) (Figure 4b). In the former category, brain BMECs are seeded on top of the Transwell insert, while other cells can be cultured on the underside of the Transwell insert (Appelt-Menzel et al., 2017). Conversely, in the latter option, the other cell types are cultured in the surrounding well (Linz et al., 2020; Booth and Kim, 2012; Carton and Malatesta, 2022). Generally, contact co-cultures produce higher TEER values compared to non-contact co-cultures (Blanchard et al., 2020; Kulczar et al., 2017).

As with monoculture Transwell models, several parameters can be modified, giving large variation between different models used in different studies. For example, different pore size can be used (0.4–3.0 μm), thus allowing for different rates of cell extravasation and direct contact between cell types (Jackson et al., 2019). A study by Stone et al., utilising three and 4 cell co-culture Transwell models, observed increased barrier integrity as measured by TEER in 12 well plates compared to 24 well plates and in 3.0 μm pore membranes compared to 0.4 μm membranes (Stone et al., 2019). For these 2D Transwell systems, the critical selection of cells and insert filters greatly influences the overall effectiveness and interpretation of the model (Chaulagain et al., 2023).

### 5.3 Limitations of 2D BBB models

The 2D Transwell model is commonly used by researchers because it is effective for investigating BBB integrity and drug transport simply and cheaply. An advantage of these models is that several methods of assessing BBB integrity can be easily incorporated into Transwell models. As seen in Figure 3a, TEER can be incorporated into Transwell models using chopstick electrodes placed in the luminal and abluminal compartments to detect the resistance to an applied electrical current, which reflects the integrity of the cell barrier formed. Additionally, paracellular permeability can be easily measured by adding tracer substances to the upper compartment and monitoring changes in their concentration between the compartments over time (Figure 3b). The monoculture Transwell BBB model (Figure 4a) is one of the simplest *in vitro* BBB models, associated with low costs and labour time, making it ideal for scaling up into high-throughput screens of drug candidates for barrier permeability (Chaulagain et al., 2023; Sivandzade and Cucullo, 2018; Carton and Malatesta, 2022). Initial monoculture Transwell BBB models were extremely limited in recapitulating the *in vivo* scenario, lacking appropriate TEER values, apparent permeability and expression of BBB specific proteins such as ABC transporters. This limitation has been overcome somewhat by the development of co-culture models. The co-culture of brain BMECs with other cells of the NVU increases the integrity and validity of the BBB model, allowing for closer mimicking of the *in vivo* setting and observation of cellular crosstalk (Sivandzade and Cucullo, 2018; Stone et al., 2019; Nakagawa et al., 2009). Despite the increased cost and time associated with optimising a multi-cellular model, co-culture Transwell models are still considered cost-effective, scalable, time-efficient, easy to use and suitable for high-throughput screening (Williams-Medina et al., 2021; Jackson et al., 2019; Stone et al., 2019).

One of the major challenges with 2D BBB models is the high variability between models from study to study. One source of variability is the cells used in the model, as mentioned in Section 3, making it challenging to compare between studies and to standardise pre-clinical testing of potential therapeutics for penetration and accumulation into the CNS tissue. The oversimplified, static, 2D nature of the BBB creates an additional limitation. In the body, the BBB exists in a dynamic 3D environment with CNS-specific tissue architecture and ECM, influenced by blood flow shear stress critical to BMEC differentiation and functioning (Traub and Berk, 1998; Cucullo et al., 2011). Contrastingly, 2D Transwell models show discrepancies from *in vivo* conditions, including irregular BMEC monolayer formation, greater BMECs-astrocyte distances as well as the insert width (10–20 μm) being thicker than the *in vivo* BBB basal lamina (Wilhelm and Krizbai, 2014; Carton and Malatesta, 2022). While cells in 2D co-cultured models can interact, they lack the intercellular dynamics of the BBB *in vivo* which can be recapitulated to a greater extent through the use of 3D BBB models.

## 6 Recent advances in 3D *in vitro* BBB models

### 6.1 Hydrogel-based BBB models

The first 3D BBB models to be described in scientific literature were hydrogel-based models. Hydrogels are water absorbing cross-linked polymers, which provide a 3D scaffold supporting the growth, cellular interactions and tissue architecture BBB models strive to reproduce. In the context of BBB modelling, hydrogel-based models offer key advantages, such as biocompatibility, tuneable mechanical properties and ECM-cell interactions. Through tuning the 3D environment, hydrogel-based models can more accurately reflect the mechanical properties of the brains ECM, which has a stiffness of around 1–2 kPa (Khoonkari et al., 2022; Stewart et al., 2017). In contrast, traditional cell culture plastics used in 2D models are significantly stiffer, with stiffness ranging from 2–4 GPa (Engler et al., 2006). Numerous polymer materials can be utilized to formulate hydrogels and can be classified into natural, synthetic or semi-synthetic sources as outlined in Table 2. The biomaterial used has distinct influence on the cellular phenotype and behaviour, thus properties such as stiffness, flexibility, elasticity and gelling structure such as pore size and topography must be taken into account (Huang et al., 2017).

**TABLE 2:** Various types of hydrogel polymers often used for *in vitro* BBB modelling and their associated advantages and limitations.

Hydrogel polymer	Classification	Key properties/advantages	Limitations	References
Collagen I	Natural (protein)	• Natural ECM component. Promotes NVU cell growth and attachment • Contains innate RGD peptides for integrin-mediated cell adhesion	• Commonly found in stiffer tissues such as bone rather than softer tissues such as the brain	Moxon et al. (2019), Grifno et al. (2019)
Gelatin	Natural (derived from collagen)	• Biocompatible - Supports neural and endothelial cell growth • Contains innate RGD peptides for integrin-mediated cell adhesion	• At physiological temperatures has poor mechanical stability • Limited tunability	Nichol et al. (2010), Guizzardi et al. (2019), Augustine et al. (2021), Ghosh et al. (2023)
Fibrin	Natural (protein)	• Biocompatible - Supports neural and endothelial cell growth • Contains innate RGD peptides for integrin-mediated cell adhesion • Promote angiogenesis	• Low mechanical strength • Low viscosity • Rapid degradation effecting stability	Benning et al. (2018), Shpichka et al. (2020), Wein et al. (2024)
Alginate	Natural (polysaccharide)	• Hydrophilic nature allows for a uniform matrix supporting cell growth and proliferation • Biocompatible, making it suitable for co-culture with NVU cells	• Must be functionalized with peptide sequences or mixed with hydrogel containing innate CAPs	Frampton et al. (2011), Lee and Mooney (2012), Khurana et al. (2018)
Polyethylene glycol (PEG)	Synthetic	• Flexible, biocompatible, biodegradable, tuneable, porous, inexpensive, hydrophilic • Rapid gelation • Stable	• Must be functionalized with peptide sequences or mixed with hydrogel containing innate CAPs	Ahmad et al. (2024), Day et al. (2018)
GelMA (gelatin methacrylate)	Semi-synthetic	• Allows for UV cross-linking and therefore precise control over machinal properties of the hydrogel • Contains RGD ligands for cell attachment	• Limited mechanical strength • Rapid degradation rate	Kim et al. (2020), Young et al. (2023)
Matrigel	Natural (mixture of ECM proteins)	• Mimics natural BM of the BBB. • Supports cell adhesion, differentiation • Gelation at physiological temperatures • Enhanced TJ expression	• Batch-batch variability • Rapid disintegration/degradation rate	Patel and Alahmad (2016), Kim et al. (2022b), Koh and Hagiwara (2024)

Collagen is a major component of the ECM, with collagen I being the most abundant type. Although it is often found in stiffer tissues like bone, with collagen IV being more abundant in soft tissues such as the brain, it remains a widely used hydrogel for 3D *in vitro* BBB model. This is due to lower cost compared to collagen IV, its tunability to brain stiffness by adjusting concentration or cross-linking mechanism, and its ability to promote NVU cell growth and attachment (Moxon et al., 2019; Potjewyd et al., 2021). In addition to collagen I, Matrigel ™ is a widely used, commercially available mixture of ECM isolated from murine sarcoma BM, known to facilitate cellular adhesion, growth, migration and invasion (Passaniti et al., 2022). Matrigel consists of laminin, collagen IV, nidogen, heparan sulphate proteoglycans and growth factors which play a role in cell adhesion and differentiation, support, stability, cell signalling and cell proliferation (Kleinman et al., 2018; Wang et al., 2020).

Alternatively, synthetic hydrogels can be generated such as polyethylene glycol (PEG), to form a polymer mesh with a water content, that is highly tuneable in terms of mechanical and topographical properties. This provides an advantage in accurately mimicking the physical and mechanical properties of brain ECM, however, the lack of naturally occurring ECM signalling cues to cells limits the physiological relevance and may hinder cell adhesion viability, differentiation or function (Caliari and Burdick, 2016). This can be tackled by “functionalising” hydrogels, wherein functional cell adhesion peptides (CAPs) which mimic the biological ligands of the ECM are incorporated, such as the tripeptide sequence Arg–Gly–Asp (RGD) and the pentapeptide sequence Ile–Lys–Val–Ala–Val (IKVAV (137,142). These peptides facilitate interaction with cell membrane receptors and cell-matrix interactions, thus more closely replicating the *in vivo* environment (Jia et al., 2016). The use of semi-synthetic scaffolds such as gelatin methacrylate (GelMA), combine the accurate tuneable properties of a synthetic hydrogel with the physiological stimulation of native ECM molecules (Li et al., 2018; Kim et al., 2020).

**FIGURE 5:**
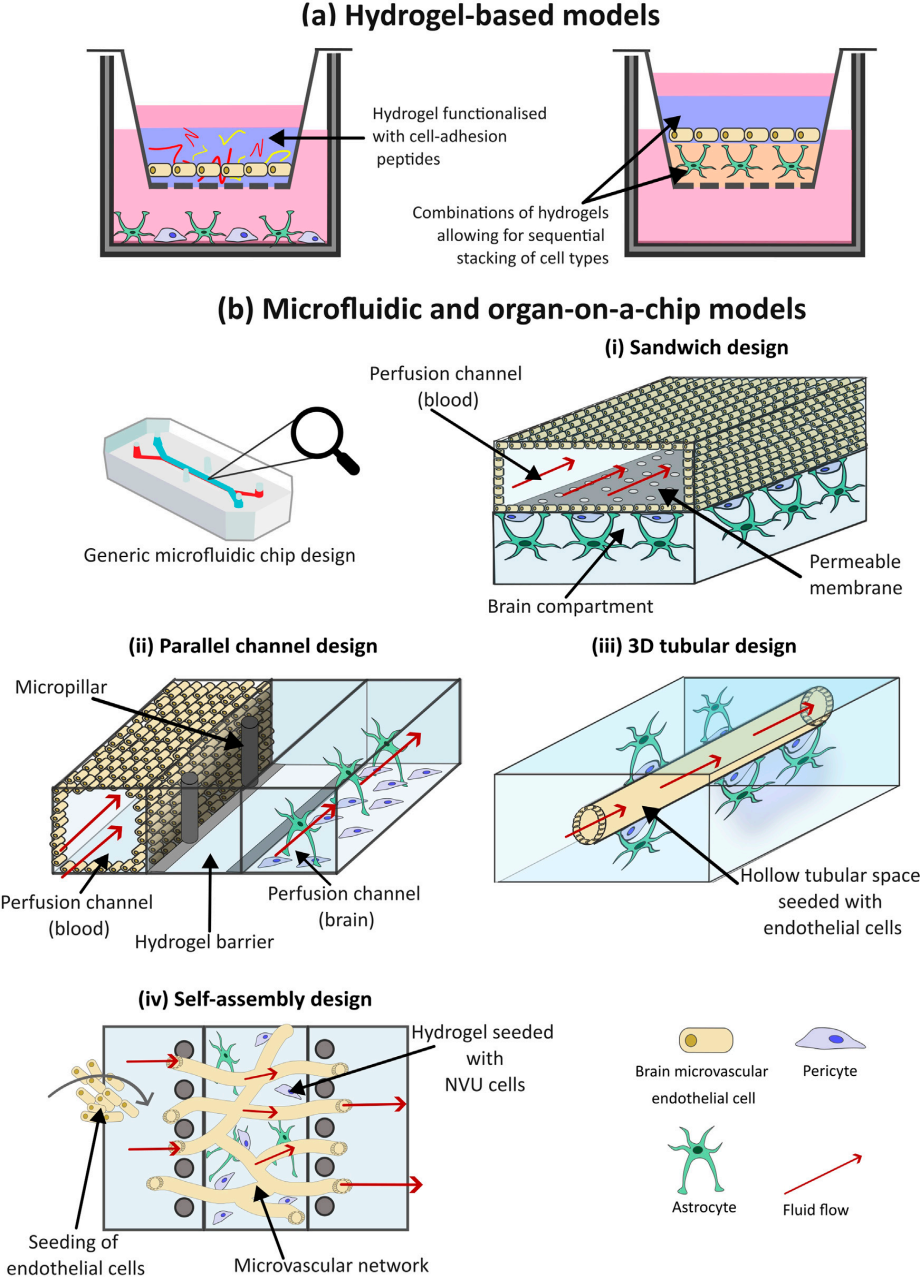
The use of hydrogels and microfluidics for three-dimensional (3D) *in vitro* models of the blood-brain barrier (BBB). **(a)** Hydrogel-based models of the BBB incorporate water-absorbing polymers, functionalized with cell-adhesion peptides, that resemble the native extracellular matrix (ECM) of the brain, to support the growth of neurovascular unit (NVU) cells within a 3D environment. **(b)** Microfluidic BBB models incorporate fluid flow to mimic the shear stress exerted by blood flow within the brain. There are multiple microfluidic configurations that can be utilized. (i) The sandwich model is the simplest of these deigns, with an upper and lower chamber separated by a porous membrane. Brain microvascular endothelial cells (BMECs) are often cultured within the top chamber, and additional NVU cells in the bottom, fluid can then flow through the channels to simulate blood flow. (ii) Parallel deign models are composed of two or more parallel chambers often separated by microchannels which allows for increased cell-cell contact compared to the sandwich model. (iii) 3D tubular designs resemble the structure of the blood vessels within the brain, for this hydrogel is moulded around a needle prior to the needle’s removal, leaving a hollow chamber for BMEC cell seeding. Additional NVU cells are seeded within the surrounding environment, representing the abluminal side of the BBB. (iv) The self-assembly design aims to mimic the branched structure of the brain microvascular. Multiple NVU cell types embedded within a hydrogel matrix are introduced to a central chamber, where they self-assemble into a BBB structure. The central channel is often flanked by two channels which provide nutrients to the cells.

Hydrogels can be used selectively in 3D BBB models to produce bespoke configurations, recapitulating the physiological structure of the BBB. As seen in Figure 5a, BMECs, either alone or with other NVU cells, can be embedded within the hydrogel scaffold. For instance, Agathe et al., developed a model incorporating immortalized human endothelial cells, astrocytes and pericytes, embedded within a hydrogel of fibronectin and collagen. In comparison to a 2D co-culture, this 3D model exhibited significantly higher expression of TJ proteins such as CLDN-5, demonstrating the enhanced physiological relevance of 3D models compared to 2D ones (Agathe et al., 2020). However, this configuration lacks an open luminal side, limiting it application in drug screening, where crossing into luminal space is required to test its penetrable abilities. In an alternative set up, endothelial cells can be seeded on top of a hydrogel structure with other NVU cell types embedded within the gel-like structure (Sreekanthreddy et al., 2016). Recently, Ahmad et al., developed a 3D *in vitro* model with normal human astrocytes embedded in a PEG matrix functionalized with adhesion peptides RGD and IKVAV, and matrix metalloproteinases sensitive cross-linking peptides, to better mimic *in vivo* ECM. Human Aortic Endothelial Cells were then seeded on top to mimic the vascular lining. The model showed higher TEER, lower Evan’s Blue permeability, and elevated ZO-1 expression within the endothelial cells, compared to 2D controls, indicating improved replication of key BBB properties (Ahmad et al., 2024). Advantages of the 3D hydrogel model were also seen in an assessment using primary human endothelial cells and astrocytes in a GelMA hydrogel, which demonstrated higher TEER values and increased expression of TJ proteins than corresponding 2D models (Saliba et al., 2025). It is evident that the inclusion of a hydrogel scaffold can increase the cell-cell contact and support for cells, thereby increasing the integrity of the barrier model and improving their translational relevance.

Despite these advantages, the choice of hydrogels which are easiest to work with may limit the accuracy of *in vivo* representation. The ECM of brain tissue is highly specialized and varies substantially from other organs with a lack of fibrillar collagen I and enrichment in the glycosaminoglycan hyaluronic acid, leading to the soft, low stiffness consistency of the brain (Lam et al., 2019; Ge et al., 2024). As mentioned in Section 1, the BM of BMECs consists mainly of laminin, collagen IV, nidogen, and heparan sulphate proteoglycans (Xu et al., 2019; Lau et al., 2013). Thus, commonly used hydrogels like Matrigel and collagen I, do not accurately represent the BBB microenvironment. Models incorporating BBB-specific ECM components, particularly mixtures of collagen IV and fibronectin, demonstrate that these substrates provide essential signalling cues that promote the formation of functional BMEC monolayers *in vitro* and support superior growth and maintenance of BMECs relative to other ECM proteins (Katt et al., 2018). The use of decellularized native brain ECM has also shown to increase the interaction between neuronal cells and supportive glial cell, aiming in the formation of functional networks in both 2D and 3D (Lam et al., 2019). Thus, in building accurate 3D models of the BBB choice of scaffolding biomaterial is critical.

### 6.2 Microfluidic and organ-on-a-chip (OOAC) devices

Initial 3D models utilized culturing in static conditions, often with cell types layered within a scaffold. However, static models methods introduce some caveats compared to the dynamicity seen *in vivo*, as they exhibit low expression of transporters normally found in the BBB, short-term viability, as well as a high permeability of normally impermeable molecules such as ions and large proteins such as antibodies (Wang et al., 2016). Microfluidic devices introduce sheer stress forces by simulating blood flow, and usually consist of a printed or casted microfluidic chip, produced from polymer materials such as polydimethylsiloxane (PDMS). Microfluidic models can vary in complexity, from simple unicellular flow circuits to complex microphysiological systems (MPS) and organ-on-a-chip (OOAC) devices that replicate one or more aspects of an organ’s *in vivo* dynamics, functionality, structure and physiological response. Exact definitions are often used interchangeably (CEN and CENELEC, 2024).

There are various configurations a microfluidic BBB model can take as demonstrated by the variety of published studies (Ceccarelli et al., 2024; Duong et al., 2021; Wang et al., 2017; Ahn et al., 2020; Xu et al., 2016; Vatine et al., 2019; Yu et al., 2020; Ohbuchi et al., 2024), and illustrated in Figure 5b. The simplest of these is the sandwich design. The set up of this model is analogous to the Transwell, comprised of two chip layers, simulating a “blood” and “brain” side. Open channels lie within the layers separated by a porous membrane. BMECs are often cultured within the top channel and supporting NVU cell types such as pericytes and astrocytes in the bottom. Prior to cell seeding, the channels can be coated with various hydrogels to enhance cell attachment. Although cell types can be layered in such a configuration, early and low complexity microfluidic models are limited in how much of the 3D microenvironment is captured.

To emulate shear stress created by blood flow *in vivo*, fluid flow can be applied to the channels of microfluid models (Vetter et al., 2025; Choi et al., 2024). Recently, Liang et al., developed a sandwich-type *in vitro* BBB model, consisting of two channels separated by a cultured hCMEC/D3 cell barrier layer, to monitor the passage of L-dopa across the BBB. Fluid flow was introduced to the upper channel, simulating blood flow, while an electrochemical sensing system was incorporated into the bottom channel, to monitor real-time movement of L-dopa across the cultured barrier. They found that ZO-1 showed greater expression under flow culture compared to static (Liang et al., 2024). This study, along with others have shown that the shear stress generated by fluid flow helped to regulate endothelial cell orientation, morphology and function (Cucullo et al., 2011; Liang et al., 2024; Ye et al., 2014). Furthermore, the incorporation of fluid flow within BBB models has been shown to affect glycocalyx gene expression. A study by Santa-Maria et al., found that compared to static conditions, incorporation of fluid flow into their BBB model resulted in upregulation of glycocalyx genes, resulting in a denser, more negatively charged glycocalyx, better representing that found *in vivo* (Santa-Maria et al., 2021).

Through the incorporation of fluid flow, sandwich models offer increased physiological accuracy, while also maintaining a simple, cost-effective approach to BBB modelling and permeability testing of potential drugs targeting CNS disorders (Booth and Kim, 2012; Wang et al., 2017). However, the configuration of these models can limit direct cell–cell contact, as influenced by the pore size of the separating membrane.

An alternative setup promoting cell contact is the use of parallel channel designs (Figure 5bii). In this configuration, two or more channels are aligned within the same horizontal plane, usually separated by microchannels, gel barriers, or micropillar arrays. Cells can be grown directly on channel surfaces or embedded in a hydrogel matrix to support 3D cultures. (Adriani et al., 2017; Cai et al., 2022). A notable example is the OrganoPlate, which can be used to accommodate up to 40 BBB models/chips per plate (Trietsch et al., 2013; Wevers et al., 2018). Each chip typically includes a three-channel setup, allowing different cell types in each channel to replicate BBB structure and function. Wevers et al. used the OrganoPlate to model the BBB for high-throughput assessment of barrier function and antibody transport (Wevers et al., 2018). The middle channel was filled with collagen I gel; Ty10 endothelial cells were seeded into the top chamber, and astrocytes and pericytes into the bottom. Fluid flow was simulated by placing the plate on a rocker. The model showed strong barrier function, low permeability and effective TJs. The main advantage of parallel channel designs over sandwich models is increased cell-cell contact. Additionally, they are less complex and time-consuming to generate, as they can be created as a complete unit. However, transport experiments can be more difficult, and TEER measurements less straightforward, as the barrier margin is not as clearly defined as in sandwich configurations.

Although the aforementioned designs incorporate fluid flow to generate shear stress; the resulting distribution does not align with physiological distributions due to the rectangular shape of the models and their flat 2D surface, which does not capture the natural 3D structure of brain capillaries. Intricacies in 3D channel designs to resemble the shape of blood vessels can help to enhance the accuracy of microfluidic models. This can be achieved by moulding a hydrogel around a wire or needle to create a hollow channel onto which endothelial cells are seeded, then perfused with media. Pericytes and astrocytes are then introduced to the abluminal side of the channel or mixed within the hydrogel prior to moulding (Figure 5biii) (Seo et al., 2022; Faley et al., 2019). However, this approach produces only straight, unbranched, uniform channels, which do not accurately replicate the complex branching structure of neural blood vessels (Dolgin, 2025). 3D printing has emerged as a potential approach for branched channel fabrication, wherein 3D hydrogel networks and NVU cells can be bio-printed into a vessel-like tube or branched formation and perfused to apply physiological shear stress (Liu et al., 2020; Paone et al., 2024). Although an improvement from sandwich and parallel designs in replicating the neural vasculature and creating a true 3D construction, the resolution of printed tubes remains a limitation. Additionally, there is challenges with bio-ink material that is simultaneously printable, biocompatible and supportive of multiple cell types. Finally, the process, material and equipment needed to fabricate a 3D bio printed model is costly and time-consuming (Mancuso et al., 2024).

A final configuration is the self-assembly design. In this model, the various cell types and hydrogel material are introduced into a single channel and spontaneously form BBB structure often via vasculogenesis, while adjacent channels provide nutrients (Figure 5biv) (Uwamori et al., 2017). Various hydrogel materials can be used for self-assembly, with fibrin being the most utilized. Other frequently used materials include Matrigel and collagen. Campisi et al., developed a model consisting of singular PDMS channel filled with cell suspension of iPSC-ECs, human pericytes and astrocytes, mixed within a fibrin hydrogel, flanked by two fluid channels (Campisi et al., 2018). The model demonstrated functional, perfusable microvasculature with selective permeability that was lower than traditional *in vitro* models and closely resembled *in vivo* measurements observed in the rat brain.

Microfluidic models are advancing constantly with a move towards the OOAC nomenclature. This model type differs from other systems which utilize planar microfluidic or hollow fibres to recreate the dynamic flow environment of the BBB by producing a simulation of the BBB *in vivo* environment and its neuronal cell types with gels or in spheroids (Moya et al., 2020). OOAC models focus on replicating the full physiological complexity of the BBB, which includes interactions between different cells of the NVU and differing environmental conditions (e.g., pathologies like AD). Microfluidic BBB models generally examine fluid dynamics and basic interactions between a few cell types. Since OOAC models incorporate ECM components, dynamic flow conditions, and multiple NVU cell types, one may deduce that this model type has a more sophisticated, complex design, even though at its basis, uses a microfluidic technique to control fluid flow and examine shear stress. OOAC models enable real-time, live monitoring of cellular interactions in an engineered *in vitro* environment and have also been utilized to view the overall complexity of other vital organs in the human body, such as the lung, liver, heart, intestines, kidneys, as well as the brain (Gonzales-Aloy et al., 2023). Early iterations of OACC models separated cell types using semi-permeable membranes. Although this allows for in-direct cell interactions via secreted molecules, direct cell-cell contact as is seen *in vivo* was missing. The syM-BBB microvasculature chip instead uses microfabricated pillars rather than a membrane, better simulating the architecture *in vivo* when BMECs, astrocytes and other supporting cells are seeded into the channels (Prabhakarpandian et al., 2013).

With precise control over microchannels, fluid behaviour, and cellular configuration microfluidic and OOAC models offer a high level of experimental control and scalability, making them suitable for high-throughput screening of potential therapeutic candidates. The reproducibility of 3D printing-based manufacturing also ensures low variability between experiments (Su et al., 2023; Casanova et al., 2024; Ferreira et al., 2024). However, some caveats exist concerning these models, as non-human cells such as rat and mouse are still widely used in these platforms, due to the low availability of human tissues for research (Phan et al., 2017). Using iPSCs to generate human BBB and NVU cells for used in microfluidic and OOAC models may overcome such limitations (Vatine et al., 2019). While BBB chip models present advantages over traditional BBB models, they also fail to fully replicate the *in vivo* BBB microenvironment due to their artificial structure and organisation. They are unable to fully capture the interaction of the BBB and circulating cells such as immune cells and their role in clearing perfused molecules like therapeutics. In addition, the gel matrix of the model often lacks a clearance mechanism similar to the glymphatic flow of the BBB, limiting their use for long-term studies (Hajal et al., 2022; Chen et al., 2018; Van Veluw et al., 2020).

### 6.3 Spheroid and organoid models of the BBB

**FIGURE 6:**
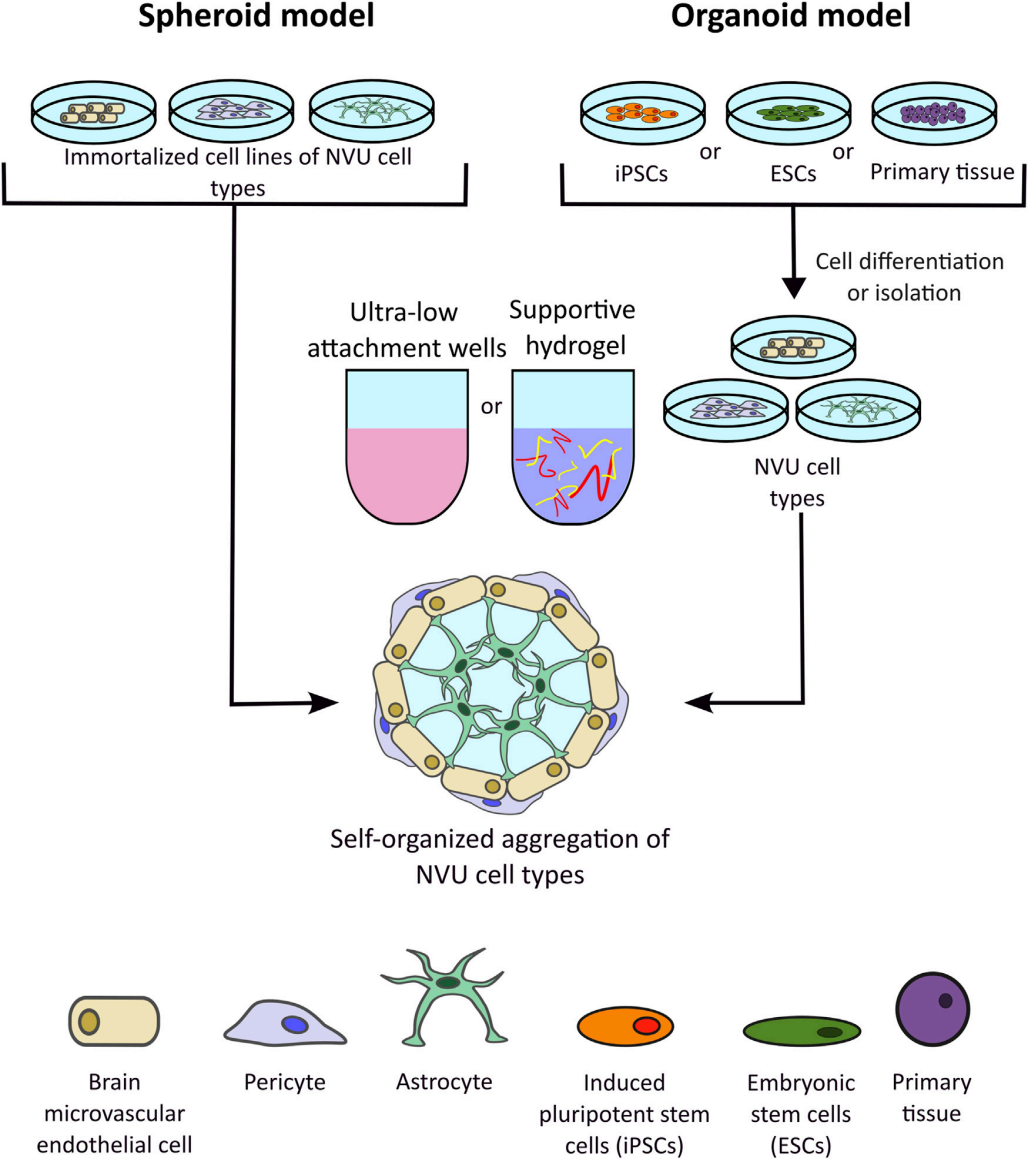
Representation of spheroid and organoid three-dimensional (3D) *in vitro* models of the blood-brain barrier (BBB). Spheroid models develop through the spontaneous aggregation of immortalised neurovascular unit (NVU) cell lines within a gel matrix or a low-attachment culture vessel. Organoid models also rely on the spontaneous aggregation of cells, however unlike spheroid models, organoids are developed from 3D cellular clusters extracted from primary tissue sources, embryonic stem cells (ESCs), or induced pluripotent stem cells (iPSCs). 3D structures can be cultures in suspension, e.g., low attachment wells, or in hydrogels.

Spheroid and organoid models are produced by the spontaneous 3D gathering of cells in a low-attachment culture vessel or within an ECM gel (Figure 6). Moreover, they are of microscale size and are structurally designed as spherical cell clusters formed through multiple methods such as single cell or co-culture techniques like hanging drop, rotating culture, or concave, ultra-low-attachment plate methods (Gonzales-Aloy et al., 2023). Initial spheroid models, for example, tumour models, consisted of a singular cell type. Now, complex co-culture spheroid models have been developed to mimic countless physiologies, including the NVU and the BBB. Although the terminology is often used interchangeably, spheroids typically consist of immortalized cell lines, whereas organoids are 3D clusters derived from primary tissues, embryonic stem cells (ESCs), or iPSCs. Organoids can self-assemble and differentiate—either spontaneously or under external stimulation—to resemble an organ in both structure and function. Organoids represent a promising avenue for personalised medicine, by taking into account patient-specific differences. Although true stem cell based-organoids have been used widely to model the brain (Eichmüller and Knoblich, 2022), the inclusion of vasculature and the BBB is rarer. More often, primary cell lines of BMECs, astrocytes and pericytes are co-cultured and allowed to self-assemble, with authors still referring to the 3D assemblies as organoids (Bergmann et al., 2018).

Recent advances have shown that spheroids have been increasingly developed to include a wider range of NVU cell types, such as microglia, oligodendrocytes, and neurons, with hopes of increasing cell-cell interactions in the *in vitro* microenvironment. Although scaffolded models provide accuracy to a supportive ECM, current trends indicate that scaffold-free methods provide effective mimics while better facilitating high-throughput screening (Gonzales-Aloy et al., 2023). Without supporting structures, direct cell-cell interactions are maintained and maximized in these models, thus causing self-organization to spontaneously occur. Compared to hydrogel-based models, which tend to utilize approximately 3 NVU cell types, spheroid 3D models can include up to five cell types such as that developed by Nzou et al. which consisted of endothelial cells, pericytes, astrocytes, neurons, microglia and oligodendrocytes and demonstrated TJ and AJs protein expression (Nzou et al., 2018). This study also allowed for functional modulation of the BBB, wherein the BBB spheroids were treated with histamine to induce the opening of the barrier, allowing the antibodies to penetrate the barrier more effectively (Nzou et al., 2018). Another scaffold-free self-assembling BBB 3D model was described by Cho et al. in which astrocytes, BMECs and pericytes were combined and found to form a selectively permeable barrier with expression of BBB associated TJ and efflux pumps, at a level more accurate to the *in vivo* than a comparable 2D Transwell system. The model was then used to screen peptides for brain penetration properties, with results showing strong agreement with *in vivo* murine model for potential hit candidates (Cho et al., 2017). A similar approach is evidenced in the published protocol from the Furihata lab, in which astrocytes, pericytes and BMECs are sequentially seeded into low-attachment wells, allowing for layered organisation and functional assessment of the barrier using dextran-dye permeability, antibody permeability and BBB transporter activity assays (Isogai et al., 2022; Kitamura et al., 2021). Selected studies have taken a truer organoid approach to developing BBB models, by combining cerebral organoids with vascular organoids *in vitro* (Dao et al., 2024; Cakir et al., 2019). One such example investigated modelling of cerebral cavernous malformations by using iPSCs derived from patient and healthy control samples to separately culture cerebral organoids and brain vascular organoids which were then merged in a functional 3D organoid assembly. These organoids recapitulated the pathophysiology of the patients, expressed BBB specific markers such as GLUT1, CLDN-5 and ZO-1 and demonstrated high TEER values of >1000 Ω cm^2^ (Dao et al., 2024).

Spheroid models have also been combined with microfluidics, to further increase biological accuracy. In a triple co-culture spheroid of astrocytes, pericytes and hCMEC/D3 cells, the spheroids demonstrated organisation with an astrocytic core, covered by pericytes and surrounded by an endothelial layer. The triple co-culture was maintained in the presence of a flowing media representing cerebral blood flow. Permeability and drug efflux assays showed that penetration of chemotherapeutics could be effectively modified by additions of mannitol (increases paracellular permeability) or verapamil (inhibits P-gp associated drug efflux) (Eilenberger et al., 2021).

Although a 3D structure offers a truer representation of the BBB environment in the body, it adds further complexity to assessing barrier integrity and transport across the barrier. As opposed to the more basic TEER or tracer dye monitoring methods for assessing permeability which can be used in 2D or microfluidic and chip models, more complicated techniques are required when assessing penetration into 3D BBB spheroids/organoids which may involve dissociation into 2D or embedding electrodes into the 3D cellular structure hence causing damage (Warschkau et al., 2022; Cakir et al., 2019). Most often confocal fluorescence microscopy for fluorescently tagged drug compounds or antibodies or mass spectrometry imaging for small molecular compounds was used to monitor the influx and accumulation (Bergmann et al., 2018).

## 7 Applications of 3D BBB models in cancer research

A primary application of 3D BBB models in cancer research is providing a physiologically relevant platform to study complex tumour-BBB interactions. Most often, these consist of brain or brain tumour model which also includes cells that represent the BBB. They can be used to investigate the mechanisms by which cancer cells, travel from a primary site to invade and colonize the brain, as well as how they interact with and transform the BBB into the BTB. A 3D perfusable hydrogel BBB model was developed by Vitale et al., 2020, effectively replicating the early stages of the metastatic cascade, with circulating tumour cells successfully colonizing the hydrogel matrix, highlighting a platform with the potential for studying how hemodynamic forces influence cell dissemination and colonization in secondary sites such as the brain (Vitale et al., 2020). Linville et al. developed a 3D BTB model using iPSC-derived brain endothelial cells cultured in a collagen I matrix to form perfusable microvessels along with metastatic breast cancer cells (JIMT-1-BR), introduced as single cells or spheroids, and macrophages, to simulate the tumour microenvironment (Linville et al., 2023). The model demonstrated key features of BTB disruption, including vascular degradation, endothelial loss, mosaic vessel formation, and increased immune cell adhesion and turnover. In model of GBM, McCoy et al. demonstrated how interactions between 3D spheroids and endothelial cells reciprocally promoted pro-tumourgenic behaviours including the increased migration of tumour cells and the increased vascular network formation of endothelial cells (McCoy et al., 2019). Such 3D models of cancer and the BTB allow for an insight into the mechanisms of cancer spread and growth in the CNS, a significant cause of morbidity and mortality for cancer patients.

Complex *in vitro* BTB models can also be used to accurately screen novel cancer therapeutics and new modalities to overcome the challenge of drug delivery to the brain. A microfluidic model of vascularised human GBM demonstrated that GBM-targeting nanoparticles loaded with chemotherapeutics could effectively cross the BTB and accumulate in tumour cells. This 3D *in vitro* model also showed strong concordance when the nanoparticles were tested in mouse models further emphasising the models validity and utility in screening novel brain cancer therapies (Hajal et al., 2022; Straehla et al., 2022). A similar approach was taken by Tricinci et al., to design a 3D printed microfluidic device, that allowed triple co-culture of BMECs, astrocytes and GBM spheroids at a 1:1 scale, forming a selective barrier through which the penetration of chemotherapy-loaded nanocarriers was monitored (Tricinci et al., 2020).

3D models of the BBB in the context of cancer also allow for understanding of drug resistance mechanisms. Regardless of the role of the BBB, modelling brain malignancy in a 3D setting has proven essential in mimicking the innate resistance to therapies seen *in vivo*. This is particularly evident in the response of GBM cell lines to the chemotherapy temozolomide, which shows markedly reduced potency in tumour spheroids compared to 2D setups (Stadler et al., 2015; Musah-Eroje and Watson, 2019; Maity et al., 2025). This phenomenon was demonstrated in a 3D BTB model where GBM spheroids were combined with primary human BMECS, pericytes and astrocytes to create a perfusable BBB, whose permeability was altered by the presence of GBM spheroids (Lam et al., 2023). Again this study showed that temozolomide had reduced effect in 3D GBM cells compared to 2D GBM, but further, very interestingly showed that the addition of a BBB led to temozolomide having no effect on the growth of GBM tumour cells, effectively blocking its cytotoxic action (Lam et al., 2023). In an advancement to this model, the authors also used it to investigate the utility of CAR-T cell treatment in GBM, a newer cancer treatment modality whose utility in solid cancers has yet to be understood (Gordon et al., 2025).

The BBB adds a unique aspect to the tumour microenvironment that must be considered in the biology, prognosis and treatment of brain tumours, both primary and metastatic. The understanding of the interactions between tumour cells and the NVU cells in the metastatic cascade that can be gained from 3D *in vitro* BBB models may provide important insight into preventative interventions that could spare patients from the debilitating symptoms and poor prognosis conferred if their primary tumour spreads to the CNS. Furthermore, such models allow for wide screens to be cast for novel therapeutics and delivery methods such as nano-technology which may offer solutions to the complicated treatment of poor survival brain cancers such as GBM and diffuse midline glioma.

## 8 Applications of 3D BBB models in non-cancer research

Outside of the oncology sphere, 3D models of the BBB can inform the aetiology, pathophysiology and treatment of neurodegenerative conditions and other CNS-associated illness. As opposed to cancer, where *in vitro* models are widely available and easy to culture, and surgical removal of tumours provides an avenue to access and analyse human tissue, CNS disorders are challenging to study in humans, with tissue availability often limited to post-mortem samples. Thus, innovative pre-clinical models of neurodegenerative disorders are essential to both understand the early stages of disease and develop new treatments.

In a general approach, 3D BBB models have been modified to study neuroinflammation by including chronic exposure to known triggers such as TNF-α or lipopolysaccharide (LPS). This method has been utilised in both simpler 2D and intricate 3D BBB models. The 3D tubular microfluidic design was employed by Herland et al. which cultured BMECs with pericytes or astrocytes in a surrounding collagen gel. Inflammation was the induced by prolonged exposure to TNF-α, with a fascinating insight that response in the form of secretion profiles was dependent on the NVU accessory cell present in the 3D model (Herland et al., 2016). 3D tubular microfluidic design of the NVU was advanced further by Seo et al. with the most complex model found in our reporting of 7 NVU associated cell types: BMECs, astrocytes, pericytes, neurons, oligodendrocytes, microglia and neural stem cells, embedded in a collagen gel (Seo et al., 2021). The addition of NVU cells showed a protective effect in preventing the increase in BBB permeability induced by LPS-associated inflammation, providing a new insight to older research that has demonstrated the role of LPS in BBB breakdown that was investigated in monoculture BMEC models (Banks et al., 2015). This work showed the contribution of many cell types to BBB maturation and function (Seo et al., 2021), demonstrating the importance of these multi 3D models to more accurately recapitulate the scenario within the human body.

OOAC technology has been used to model the pathology of Parkinsons disease (PD) by combining dopaminergic neurons, astrocytes, microglia, pericytes, and microvascular brain endothelial cells, representing the substantia nigra area of the brain which is affected in PD (Pediaditakis et al., 2021). The synucleinopathy associated with PD was induced by the addition of exogenous human recombinant αSyn monomers fibrils into the channels of the microfluidics device. These modifications allowed for recapitulation of *in vivo* PD features including phosphorylated αSyn, mitochondrial impairment, neuroinflammation, and compromised barrier function, as measured by dextran and lucifer tracers (Pediaditakis et al., 2021).

Several 3D models have also assessed the role of the BBB in AD. In one self-assembled 3D model, NVU cells were combined with stem cell-derived neurons and astrocytes harbouring Familial AD (FAD) mutations. With the addition of microfluidic flow, this model was capable of maintenance over 30 days and showed the induction of AD pathology increased BBB permeability and caused dysregulation of key endothelial and pericyte expression (Pavlou et al., 2025). Furthermore, the model allowed for visualisation and measurement of amyloid-beta (Aβ) accumulations. In a similar microfluidics model, human neural progenitor cells transfected with FAD mutations were cultured in a Matrigel scaffold then attached to a layer of BMECs, separated by a collagen I layer. Again, this model demonstrated that the presence of AD-phenotype neural cells triggered an increased in BBB permeability, which the authors lined to decreased expression of CLDNs and cadherin genes. Furthermore, the accumulation of Aβ could be seen in at the BMEC layer (Shin et al., 2019). An OOAC of diabetes mellitus in AD, illustrated that the link between diabetes and AD may be related to downregulation of sirtuin 1, showing the importance of 3D multicellular models in facilitating the understanding of co-morbidities and complex CNS disease manifestations (Jang et al., 2022). OOAC AD models have also been used to investigate RMT mediated targeted drug delivery, such as gold-nano rods functionalised with angiopep-2, allowing transportation across the barrier by LRP1 on BMECs, and delivery of a peptide designed to prevent Aβ accumulations (Palma-Florez et al., 2023).

3D spheroid models of the BBB have also been combined with patient derived materials to model disease manifestation, for example, in the study by Caratis et al. who applied CSF from patients with MS to their tri-culture BBB spheroids. This translational method revealed a key inflammatory pathway activated by the MS disease phenotype which decreased TJ associated proteins and promoted the migration of proinflammatory immune cells (Caratis et al., 2025). Such an approach overcomes the difficulty in obtaining human brain tissue for research by combining immortalised cell lines with cerebral spinal fluid and could be applied to other disease manifestations.

Organoid-type models have allowed for the modelling of precise CNS dysfunction, such as the consequences of ishemic stroke (Wang et al., 2021; Wei et al., 2023). Wang et al. demonstrated that primary neural stem cells, differentiated into NVU cells and BMECs can self-assemble in a Matrigel scaffold. The authors then simulated the oxygen and glucose deprivation seen in cerebral ischemia and saw neurovascular damage comparable to that seen *in vivo*, including disaggregation of vascular structure and reduction in TJ number. Treatment with VEGF was then seen to prevent this damage, further demonstrating the value of the model in both understanding the physiology of neurovascular damage and assessing preventative treatment modalities (Wang et al., 2021).

Collectively, these studies highlight the versatility of 3D BBB models in elucidating disease mechanisms beyond oncology, enabling the recreation of complex neurovascular environments that are otherwise inaccessible in human patients. By incorporating multiple cell types of the NVU, disease-specific conditions, and patient-derived materials, such models bridge critical gaps between *in vitro* experimentation and *in vivo* pathophysiology.

## 9 Reproducibility, regulatory relevance, and standardization of BBB *in vitro* models

The development and use of 3D *in vitro* models aligns with the “3Rs (Replacement, Reduction, Refinement)” principles, which is an ethical framework protecting the welfare of animals in research by promoting the use of alternative pre-clinical models (Graham and Prescott, 2015). Compared to animal models, proponents of the “3Rs” suggest 3D *in vitro* methods offer increased cost-effectiveness, reproducibility and ease of transition into a clinical environment (Huang and Gao, 2018). Furthermore, considering the difficulty in bringing novel therapies to market, there is a strong impetus at present to find alternatives to animal models. Food and Drug Administration (FDA) clinical approval rates of new therapies has reported to be as low as 6% (Dowden and Munro, 2019), with CNS therapies representing the second highest proportion of phase II and III clinical trial failures (Harrison, 2016). Much of this failure has been attributed to the inaccuracy of non-human animal models. As such, the FDA recently announced plans to phase out animal testing of therapies, in favour of real-world-data, computational models and complex *in vitro* 3D organoid and OOAC models (Food and Drug Administration, 2025).

Further adoption of *in vitro* models into the general pipeline of drug regulation can be limited by the translational relevance and accuracy from *in vitro* to *in vivo*. For BBB models, however, several studies have demonstrated strong agreement between *in vitro* findings and *in vivo* or human studies. For example, a human iPSC based Transwell model developed by Le Roux et al. showed good correlation for a number of tested drugs to CNS accumulation in human brains, as measured by brain Positron Emission Tomography (PET) imaging (Roux et al., 2019). On the computational side, the FDA’s Division of Applied Regulatory Sciences (DARS) have developed quantitative structure-activity relationship (QSAR) models to predict BBB permeability of drug compounds with the aim of reducing the use of *in vivo* testing. As such, BBB modelling may be an excellent avenue for increasing the utility of *in vitro* and in sillico testing and reducing the reliance on animal models in drug regulation (Faramarzi et al., 2022).

*In vitro* BBB models are used in the early stages of novel drug development, but are usually limited to basic Transwell models, using easy to grow but non-brain cells such as Madin-Darby Canine Kidney (MDCK) or Caco-2 or non-human cells (Table 3). There is no standardized testing protocol designated for the pre-clinical testing of CNS targeting agents. There has been commercialisation of BBB model services (Blood-Brain Barrier, 2025) and ready to buy kits (ScienCell, 2025) which may go some way to standardisation in the field. At present, these include Transwell-type assays and microfluidic chips such as those sold by Syn-Vivo (Prabhakarpandian et al., 2013; SynBBB, 2025). Despite this, reproducibility between studies is challenging due to the sheer number of technical variations, for example, batch variability in ECM coatings, differences in cell expression profiles. 3D models, in particular spheroids and organoids, while in increasing physiological relevance, also increase structural and functional heterogeneity, making comparative analysis challenging. Although BBB models using human iPSC-derived cells are advancing this field, their use is not yet widespread or standardized enough to replace animal models in regulatory pipelines due to their natural variability and sensitive differentiation approaches (Piergiovanni et al., 2021).

**TABLE 3:** Summary of BBB model types and comparison of advantages, limitations, reported trans-epithelial electrical resistance (TEER) readings and recommended best usage.

Model type	Key features	Advantages	Limitations	TEER (Ω·cm^2^)	Best use cases	References
Hydrogel-Based Models	3D scaffolds using natural, synthetic, or semi-synthetic hydrogels (e.g., collagen I, Matrigel, PEG, GelMA)	• Biocompatible • Tunable stiffness and porosity • Supports NVU cell growth • Functionalized hydrogels improve cell interaction	• Static, lacks fluid dynamics • Common hydrogels may not mimic native brain ECM. • Incomplete immune/glymphatic interaction • Gel may hinder measurements of barrier integrity	∼50 to ∼800	• Cell-ECM interaction studies • ECM composition effects • Early-stage BBB development models	Potjewyd et al. (2021), Agathe et al. (2020), Sreekanthreddy et al. (2016), Ahmad et al. (2024), Saliba et al. (2025), Augustine et al. (2021), Singh et al. (2023)
Microfluidic/Organ-on-a-Chip (OOAC)	Dynamic models incorporating fluid flow and shear stress in microchannels or hollow/3D printed vessels	• Mimics blood flow • Shear stress capabilities: ∼0.01 dyn/cm^2^ to ∼10 dyn/cm^2^ • Real-time monitoring • Scalable and reproducible • Compatible with high-throughput assays • Can simulate pathological conditions • Measurements of barrier integrity can be incorporated into chip design	• Technical complexity • Limited direct cell-cell contact in some designs • Incomplete immune/glymphatic interaction • Requires specialized materials/equipment	∼20 to ∼4000	• Drug permeability testing • Disease modelling • Transport mechanism studies • High-throughput screening	Seo et al. (2022), Ceccarelli et al. (2024), Duong et al. (2021), Wang et al. (2017), Ahn et al. (2020), Xu et al. (2016), Vatine et al. (2019), Yu et al. (2020), Ohbuchi et al. (2024), Choi et al. (2024), Liang et al. (2024), Santa-Maria et al. (2021), Trietsch et al. (2013), Liu et al. (2020), Paone et al. (2024), Moya et al. (2020)
Spheroid/Organoid Models	Scaffold-free or ECM-embedded 3D cell aggregates, self-organizing, derived from immortalized cells or iPSCs	• Includes multiple NVU cell types • High physiological relevance • Enhanced direct cell-cell interactions • Potential for personalized medicine	• Difficult to standardise • Limited flow/perfusion unless integrated with microfluidics • Complex imaging/penetration analysis	TEER measurement is challenging in 3D organoids due to their enclosed architecture. Requires dissociation to 2D or embedding of electrode within the 3D cellular assembly 1,190.8 ± 106 (Dao et al., 2024) 351 ± 10 (Cakir et al., 2019)	• Cell interaction studies • Personalized BBB modelling • Neurotoxicity testing • Complex in vivo-like BBB simulation	Bergmann et al. (2018), Nzou et al. (2018), Cho et al. (2017), Isogai et al. (2022), Kitamura et al. (2021), Dao et al. (2024), Cakir et al. (2019)

The OOAC is a particular setting where strides are being made to address reproducibility and standardisation challenges. The European Commission Joint Research Centre (JRC) have identified standardization need for OOOAC models which include biocompatibility, testing methods, and material safety (Piergiovanni et al., 2021). Following this, the JRC together with the European Committee for Standardization and the European Committee for Electrotechnical Standardization (CEN and CENELEC) developed a detailed roadmap outlining future standardization recommendations (CEN and CENELEC, 2024). Critical areas highlighted in the roadmap include the harmonization of terminology, minimum reporting requirements for biological aspects such as cells, biocompatibility and sterilization standards and material characterization (CEN and CENELEC, 2024). The issue of standard terminology has also been raised by Advancing Standards Transforming Markets (ASTM) International, who have published ASTM F3570-22 which defines basic terms and presents the relationships of the scientific fields related to MPS such as OOAC systems (Advancing Standards Transforming Markets ASTM International, 2022).

The Centre for Alternatives to Animal Testing in Europe (CAAT-Europe) t4 thinktank has held regular workshops on biologically-inspired MPS since 2015 and has been setting a benchmark for assessing scientific, industrial, and regulatory trends in MPS (Marx et al., 2025). This group identified the lack of trust and validation in data derived from OOAC systems and recommend independent testing centres and closer interaction with industry stakeholders such as the International Consortium, Microphysiological Systems (IQ MPS) affiliate (Baker et al., 2024). Progress is underway, with several examples of OOAC systems being used as supporting evidence in European Medicines Agency (EMA) or FDA investigational new drug applications (Marx et al., 2025), however there is a need to define the role of OOAC data in the weight of evidence amongst regulators and establish the presence of such models at an the International Organization for Standardization (ISO) level (CEN and CENELEC, 2024). Explicit guidance for incorporating novel *in vitro* platforms in the drug development pipeline has not been released, and is assessed on a case-by-case basis, however, the EMA has published a recent recommendation that guidelines be updated to include the advancements in OOAC systems (European Medicines Agency, 2023). It is evident that issues regarding reproducibility, standardisation and use in regulatory development are a challenge for non-animal *in vitro* models, including those of the BBB, yet progress is advancing steadily, holding promise for widespread utilisation.

## 10 Discussion

This review highlights the rapid evolution and potential of 3D *in vitro* BBB models as transformative tools for investigating pathologies of the brain and advancing drug discovery. A wide spectrum of 3D BBB models has been developed, ranging from hydrogel-based scaffolds, microfluidic devices, and OOAC platforms to spheroid and organoid systems—each offering distinct strengths in replicating various aspects of BBB physiology. These models incorporate diverse cellular components of the neurovascular unit, enable dynamic environmental simulation, and allow for disease-specific investigations. Importantly, the review underscores that the design of a 3D model—choice of cell types, ECM scaffolding materials, spatial configuration, and inclusion of shear stress—greatly influences its biological relevance and applicability to specific research questions, whether mechanistic, therapeutic, or translational. The different modalities also open 3D BBB models towards the possibilities for personalized medicine to test patient specific drug regimens, concentrations and combinations.

Utilizing advanced biocompatible materials and tissue engineering techniques, 3D *in vitro* models better mimic the structural and functional properties of the BBB, allowing for a more accurate platform to study CNS disease pathology and treatment (Chen et al., 2021; Walters-Shumka et al., 2023). 3D BBB models present significant advantages compared to 2D models, the most prominent of these being increased physiological relance to the *in vivo* spatial organisation of the BBB. 3D models also support increased cell-cell and increased cell-ECM interactions among NVU cells (Stukel and Willits, 2018; Nzou et al., 2018). As a result, they showcase more accurate barrier properties, such as increased TJ expression, a crucial characteristic contributing to BBB function. By more accurately reflecting the structure and function of the BBB, 3D models present as an improved platform for drug screening and predicting drug transport across the BBB in human patients. Importantly, accurate pre-clinical models aid in identifying promising drug candidates and eliminating ineffective compounds early, thereby reducing the risk of clinical trial failure and detriment to trial patient cohorts.

With these advantages in mind, many researchers have advanced the pre-clinical BBB model to specifically simulate brain associated pathologies such as cancer and neurodegenerative disease as discussed in this review. *In vitro* 3D models lend themselves to bespoke design and adaptability. Cell types and environmental cues can be precisely controlled to model a variety of CNS disorders. At the cutting edge of pre-clinical testing, organoid models derived from iPSCs or tissue from a patient can provide an opportunity for personalized medicine, patient specific-disease modelling and targeted treatment approaches (Xu et al., 2023).

However, the growing variety in model design described in this review, presents a significant challenge: the lack of standardized protocols limits cross-study comparability and hinders the development of universally accepted benchmarks for compound screening and efficacy assessment. While some platforms excel in mimicking barrier tightness, others better capture immune or metastatic interactions or neuro-inflammation, making model selection highly context-dependent but also potentially inconsistent across the field. A critical reflection of the current state reveals that although 3D models surpass 2D systems in physiological relevance and reduce reliance on animal testing, issues of reproducibility, scalability, and throughput remain substantial barriers to clinical translation. Each 3D BBB model type discussed has various advantages and limitations which when evaluated together reveals key differences in physiological relevance, complexity, scalability, and application (Table 3). Each model type, therefore, serves a different niche: hydrogel models excel in recapitulating the ECM structure and cell-matrix interactions but lack dynamicity; microfluidic and OOAC platforms offer dynamic flow and experimental control, ideal for mechanistic and pharmacological studies; while spheroids/organoids provide unmatched cellular complexity, mimicry of tissue-specific architecture and patient specificity.

## 11 Conclusion

Overall, this review emphasizes that no single 3D BBB model is universally optimal and utilised at present; rather, thoughtful selection and transparent reporting of model parameters are essential for maximizing their utility. Ultimately, a combination of model types or hybrid systems may offer the most comprehensive representation of the BBB, with future directions pointing toward integrated platforms that merge the physiological accuracy of OOACs with the complexity and personalization of organoids. Progress will depend not only on further technological refinement but also on collaborative efforts to establish validated, application-specific standards. By addressing these limitations, 3D BBB models can truly realize their potential in revolutionizing the study of brain pathologies and accelerating the development of effective, brain-penetrant therapies.
